# An established protocol for generating transgenic wheat for wheat functional genomics *via* particle bombardment

**DOI:** 10.3389/fpls.2022.979540

**Published:** 2022-12-08

**Authors:** Yaqiong Wang, Jian Zeng, Peipei Su, Hongyan Zhao, Li Li, Xiaoxue Xie, Qian Zhang, Ya’nan Wu, Ruibin Wang, Yufan Zhang, Boju Yu, Mingjie Chen, Yuesheng Wang, Guangxiao Yang, Guangyuan He, Junli Chang, Yin Li

**Affiliations:** ^1^ The Genetic Engineering International Cooperation Base of Chinese Ministry of Science and Technology, The Key Laboratory of Molecular Biophysics of Chinese Ministry of Education, College of Life Science and Technology, Huazhong University of Science & Technology, Wuhan, China; ^2^ Guangdong Provincial Key Laboratory of Utilization and Conservation of Food and Medicinal Resources in Northern Region, Shaoguan University, Shaoguan, Guangdong, China

**Keywords:** particle bombardment-mediated wheat transformation research topic: women in plant biotechnology 2022 wheat, genetic transformation, particle bombardment, transgenic plants, functional genomics, genetic improvement

## Abstract

Wheat is one of the most important food crops in the world and is considered one of the top targets in crop biotechnology. With the high-quality reference genomes of wheat and its relative species and the recent burst of genomic resources in Triticeae, demands to perform gene functional studies in wheat and genetic improvement have been rapidly increasing, requiring that production of transgenic wheat should become a routine technique. While established for more than 20 years, the particle bombardment-mediated wheat transformation has not become routine yet, with only a handful of labs being proficient in this technique. This could be due to, at least partly, the low transformation efficiency and the technical difficulties. Here, we describe the current version of this method through adaptation and optimization. We report the detailed protocol of producing transgenic wheat by the particle gun, including several critical steps, from the selection of appropriate explants (i.e., immature scutella), the preparation of DNA-coated gold particles, and several established strategies of tissue culture. More importantly, with over 20 years of experience in wheat transformation in our lab, we share the many technical details and recommendations and emphasize that the particle bombardment-mediated approach has fewer limitations in genotype dependency and vector construction when compared with the *Agrobacterium*-mediated methods. The particle bombardment-mediated method has been successful for over 30 wheat genotypes, from the tetraploid durum wheat to the hexaploid common wheat, from modern elite varieties to landraces. In conclusion, the particle bombardment-mediated wheat transformation has demonstrated its potential and wide applications, and the full set of protocol, experience, and successful reports in many wheat genotypes described here will further its impacts, making it a routine and robust technique in crop research labs worldwide.

## Introduction

Wheat is one of the top three crops in global cereal production and serves as the staple for over 35% population worldwide (Hazard et al., 2020). Fluctuated environments and an increased global population pose major threats to world food security and agriculture sustainability. To meet the global food demands, wheat breeding aiming at increased yield, improved quality, and better tolerance to various stresses has become more challenging. Thus, biotechnology breeding and genomics breeding have been developed to take these challenges, which need transgenic wheat as a platform technology to study gene functions and for wheat genetic improvement (Purugganan and Jackson, 2021).

Wheat was the last major cereal crop in which transformation was achieved (Vasil et al., 1992). Throughout the 1990s, wheat transformation using particle bombardment has been established with great contributions made by the scientists from Rothamsted Research (Barcelo and Lazzeri, 1995; Barro et al., 1998; He and Lazzeri, 1998; Barro et al., 1999; Rasco-Gaunt et al., 1999; He and Lazzeri, 2001; He et al., 2001; Rasco-Gaunt et al., 2001; Pastori et al., 2001; Sparks and Jones, 2004; Goodwin et al., 2005). *Agrobacterium*-mediated transformation of wheat has been developed later with further adaptation and optimization to achieve higher efficiency (Jones et al., 2005; Shrawat and Loerz, 2006; He et al., 2010; Richardson et al., 2014; Wang et al., 2016, Wang et al., 2017). The particle bombardment- and *Agrobacterium*-mediated transformation approaches have distinct features with respective advantages and disadvantages (see Discussion).

Recently, high-quality reference genomes have been made available in several major *Triticum*/*Aegilops* species, including common wheat (*Triticum aestivum* L.), wild emmer wheat (*Triticum turgidum* spp. *dicoccoides*), durum wheat (*T. turgidum* spp. *durum*), and several diploid Triticeae species (Avni et al., 2017; Luo et al., 2017; Zhao et al., 2017; International Wheat Genome Sequencing Consortium (IWGSC), 2018; Ling et al., 2018; Maccaferri et al., 2019; Li et al., 2022). Other genomic resources have been rapidly accumulated as well, pushing forward wheat genetics to the post-genomics era (Ramírez-González et al., 2018; Cheng et al., 2019; Juliana et al., 2019; Pont et al., 2019; Chen et al., 2020; Pang et al., 2020; Sansaloni et al., 2020; Walkowiak et al., 2020). The huge volume of genomic knowledge enables faster identification of functional genes or variants in wheat. Thus, the demands for functional studies of genes have become unprecedented in wheat, accordingly requiring the production of transgenic wheat to be a robust and routine technique.

Gene-editing technologies, while developed more recently, can precisely manipulate specific genomic sequences and has the potential to replace mutagenesis approaches in functional study and molecular breeding. The gene-editing technologies include three types, zinc finger nucleases (ZFNs), transcription activator-like effector nucleases (TALENs), and clustered regularly interspaced short palindromic repeat-associated endonucleases (CRISPR/Cas). Particularly, the CRISPR/Cas-based technologies have been expanded to allow for precise modification of genomic DNA including targeted knockout, substitution, insertion, multi-locus editing, and large fragment deletion, and have been applied to many major crops as a promising tool in molecular breeding (Gao et al., 2021; Liu et al., 2022). In wheat, targeted knockout editing of two, three, four, and five genes simultaneously has been achieved through CRISPR/Cas9-based editing, and multiple superior alleles were quickly aggregated in one generation, greatly reducing the breeding time (Luo et al., 2021). Rapid and precise editing of powdery mildew resistance (MLO) gene *Tamlo-R32* in common wheat well demonstrates the power of CRISPR-Cas technology in generating new germplasm and in speeding up the breeding process (Li et al., 2022). Genetic transformation technology also helps to promote the further development of gene-editing technology.

**Figure 1:**
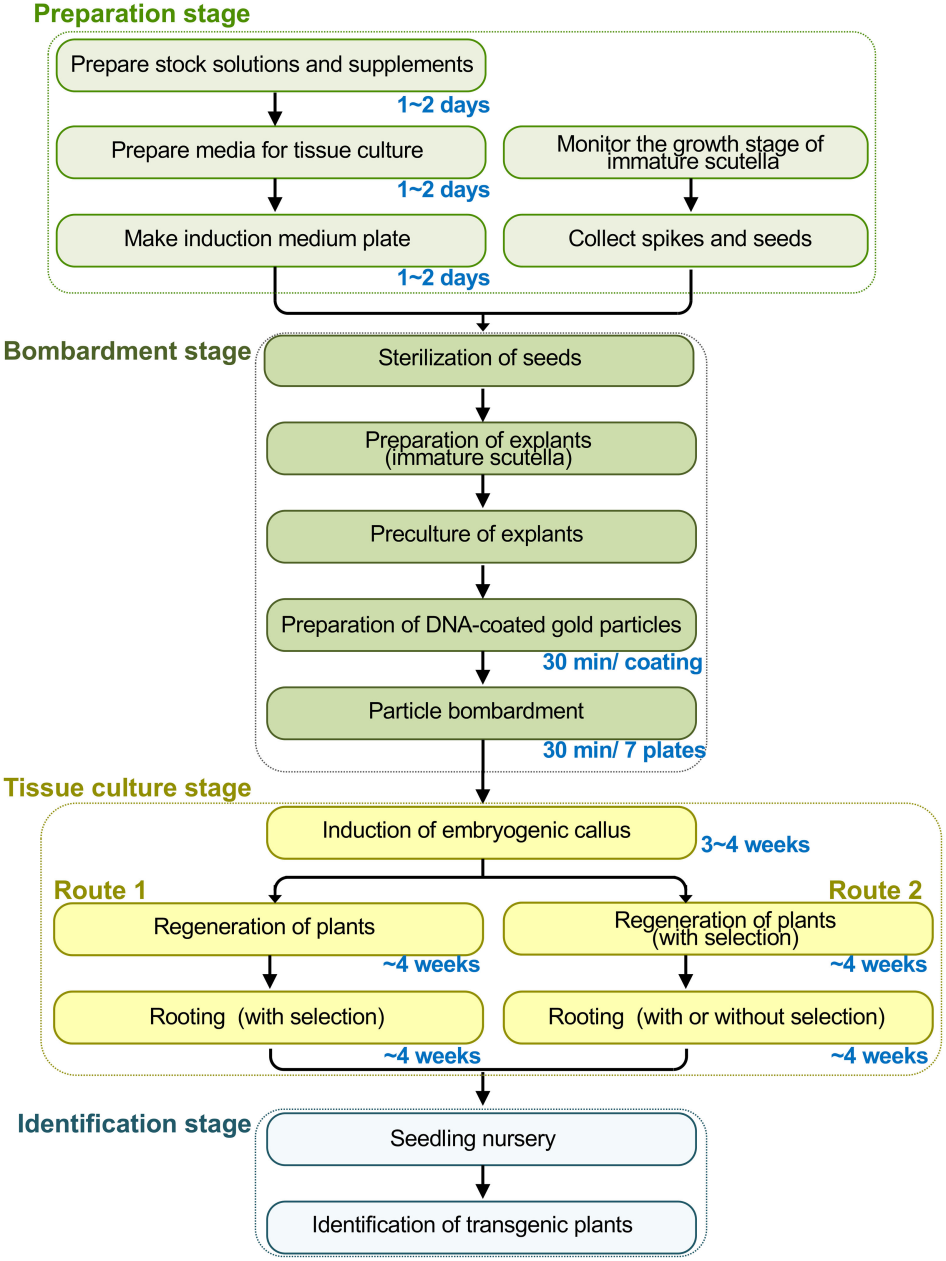
Overview of the wheat transformation process mediated by particle bombardment. The process can be divided into four stages with the time length of several key steps indicated: the preparation stage, the bombardment stage, the tissue culture stage, and the stage for identification of transgenic plants. Particularly, the selection system of *bar* gene and phosphinothricin (PPT) is described in our protocol. In the tissue-culture stage, two routes could be used to select transgenic seedlings: 1) do not add PPT in the regeneration medium and conduct selection in the rooting stage; 2) conduct selection to the calli in the regeneration stage to largely reduce the working load of tissue culture, while selection may or may not be performed during the rooting stage. Notably, after particle bombardment, a plasmolysis phase immediately before the bombardment, or a recovery culture immediately after shots, could be applied.

Here, we describe the most current form of particle bombardment-based wheat transformation. This protocol has been adapted from the previous methods and augmented with many technical details based on our lab’s extensive experiences with wheat transformation (Sparks and Jones, 2004, Sparks and Jones, 2009; Jones and Sparks, 2009; Lazzeri and Jones, 2009; Tamás-Nyitrai et al., 2012; Sparks and Jones, 2014; Sparks and Doherty, 2020). Our lab has studied wheat transformation and genetic improvement for over 20 years with continuous funding from the National Major Projects of China for Genetically Modified New Varieties. The protocol is a stepwise guide covering the following sections (**Figure 1**): 1) preparation of consumables, equipment, reagents, and media; 2) preparation of explants for transformation; 3) coating gold particles with DNA; 4) particle bombardment; 5) tissue culture and selection; 6) molecular identification of transgenic plants. The present protocol uses immature scutella as explants, while it has been readily adapted for immature inflorescence (Barcelo and Lazzeri, 1995; He and Lazzeri, 2001; He et al., 2001). This protocol can use *bar* or *nptII* as the selectable marker genes with phosphinothricin (PPT) and G418 as the respective selection reagents, while other selection methods can be used (**Figure S1**).

The purpose of this protocol is to update the particle bombardment-mediated transgenic method and to distribute it widely to the wheat research community as a routine and robust technique. Additionally, we compile our results of transgenic wheat studies and those previously published by others, demonstrating that this method has wide genotype compatibility in performing transgenic wheat studies.

## Materials and equipment

### Consumables, equipment, raw chemicals, and reagents

#### Reagents for seed disinfection

The equipment and important consumables and reagents used in this method are shown in **Table 1**. In the present protocol, 0.1% mercury dichloride, 75% (v/v) ethanol, and sterile distilled water (SDW) are used for seed disinfection, while previous reports have also described other useful ways for the disinfection of wheat seeds (Wang et al., 2016; Wang et al., 2022) (**Note 1**).

**Table 1:** Equipment and important consumables and reagents used in this protocol.

Type^a^	Equipment	Manufacturer	Model/catalog no.	Purpose
E	Precision peristaltic pump	LongerPump	WT 600-2J	Filtration medium
C	Sterile capsule	Sartorius Sterdium biotech	5441307H4–OO 0.45+0.2 µm	Filtration medium
E	Ultra-clean hood	HDL Apparatus	NA^b^	Sterile experiments
E	Electronic balance	METTLER TOLEDO	NA	Weighing
E	Autoclave		NA	Autoclaving
C	1,100 psi rupture disks	Bio-Rad, USA	1652329	Particle gun consumables
C	Macrocarriers	Bio-Rad, USA	1652335	Particle gun consumables
C	Macrocarrier holders	Bio-Rad, USA	1652322	Particle gun consumables
C	Stopping screens	Bio-Rad, USA	1652336	Particle gun consumables
C	0.6 µm gold microcarriers	Bio-Rad, USA	1652262	Particle gun consumables
R	Picloram (4-amino-3,5,6-trichloropicolinic acid)	Sigma-Aldrich, USA	P5575	Supplements for the tissue culture medium
R	2,4-D	Sigma-Aldrich, USA	31518	Supplements for the tissue culture medium
R	Zeatin	Sigma-Aldrich, USA	Z0164	Supplements for the tissue culture medium
R	Glufosinate ammonium (PPT)^c^	Sigma-Aldrich, USA	45520	Supplements for the tissue culture medium
R	Spermidine	Sigma-Aldrich, USA	S2626	Reagents for coating gold particles with DNA

a E, equipment; C, consumables; R, reagents;

b NA, not applicable.

c Glufosinate ammonium is synthetic phosphinothricin (PPT) bound to ammonium and the active component in herbicides (i.e., Basta). This can be replaced with bialaphos (phosphinothricylanalylanaline, sodium) at the concentration of 3–5 mg/L in the tissue culture medium for selection purposes.

#### Stock solutions and supplements for preparing the tissue culture medium

Several stock solutions need to be prepared before the transformation (**Table 2** and the corresponding **Supplementary Tables**). After the preparation of the stock solutions, they can be stored in the corresponding conditions for over 3 months (**Note 2**).

**Table 2:** A list of stock solutions and their components for the preparation of the tissue culture media.

Stock solution	Components	Store conditionsafter sterilization	Detailed formula
10× MS macrosalts	NH_4_NO_3_, KNO_3_, KH_2_PO_4_, MgSO_4_·7H_2_O, CaCl_2_·2H_2_O	4°C	**Table S1**
10× L microsalts	MnSO_4_·H_2_O, H_3_BO_3_, ZnSO_4_·7H_2_O, KI, Na_2_MoO_4_·2H_2_O, CuSO_4_·5H_2_O, CoCl_2_·6H_2_O	4°C	**Table S2**
100× MS FeNaETA	Na_2_EDTA·2H_2_O, FeSO_4_·7H_2_O	4°C	**Table S3**
1000× MS vitamins (-Glycine)	Nicotinic acid, thiamine HCl, pyridoxine HCl	4°C	**Table S4**
25× 3AA^a^	l-Glutamine, l-proline, l-asparagine	−20°C in dark in 40 ml/200 ml	**Table S5**
100 mg/ml of inositol	Myo-inositol	4°C	**Table S6**
10× L7 macrosalts	NH_4_NO_3_, KNO_3_, KH_2_PO_4_, MgSO_4_·7H_2_O, CaCl_2_·2H_2_O	4°C	**Table S7**
200× L vitamins and inositol	Inositol, thiamine HCl, nicotinic acid, pyridoxine HCl, ascorbic acid, Ca-pantothenate	−20°C in 50 ml per aliquot	**Table S8**

a The 3AA stock solution can be replaced by using individually added single amino acid (0.75 g/L of l-glutamine, 0.15 g/L of l-proline, and 0.1 g/L of l-asparagine).

Four supplements, including 1 mg/ml of picloram, 1 mg/ml of 2,4-D, 10 mg/ml of zeatin, and 100 mg/ml of PPT, are made as follows:

**(1)** 1 mg/ml of picloram. Weigh 100 mg of picloram. Add a few drops of NaOH to dissolve. Add distilled water to a volume of 100 ml and mix well. Filter sterilize. Aliquot in 10 ml. Store in freezer (−20°C).
**(2)** 1 mg/ml of 2,4-D. Weigh 100 mg of 2,4-D. Add 100 ml of 70% ETOH. Mix well/vortex. Filter sterilize. Aliquot in 1 ml. Store in freezer (−20°C).
**(3)** 10 mg/ml of zeatin. Weigh 100 mg of zeatin. Add 1 ml of 1 M HCl to dissolve. Add 9 ml of distilled water. Mix very well/vortex. Filter sterilize. Aliquot in 1 ml. Store in freezer (−20°C).
**(4)** 100 mg/ml of PPT. Weigh 500 mg of glufosinate ammonium (PPT). Dissolve in 50 ml of distilled water. Mix very well/vortex. Filter sterilize. Aliquot in 1 ml. Store at −20°C.

#### Preparation of wheat tissue culture media

For the tissue culture process of wheat transformation, 2× MS induction medium, 2× regeneration medium, 2× selection medium, and 2× rooting medium are made as follow (**Tables 3**–**6**). The 2× medium should be stored at 4°C after filter sterilization. 2× Agargel with a concentration of 13 g/L is used for making 1× medium plate together with each of the four above-mentioned 2× medium for tissue culture. Weigh 7 g of Agargel in a 500-ml of Schott bottle and shake well before autoclaving (121°C for at least 20 min). After autoclaving again, shake well the melted Agargel while hot (**Note 3**). Store in an incubator (temperature > 50°C) to keep the 2× Agargel as a liquid before mixing it with other 2× medium (**Notes 4, 5**).

**Table 3:** The formula of 2× MS induction media.

Components	1 L	5 L
Sucrose	60 g	300 g
10**×** MS macrosalts	200 ml	1,000 ml
1,000**×** L microsalts	2 ml	10 ml
100**×** MS FeNaEDTA	20 ml	100 ml
1,000**×** MS vitamins (-Gly)	2 ml	10 ml
25**×** 3AA solution (amino acid)	40 ml	200 ml
10 mg/ml of myo-inositol	20 ml	100 ml
1 mg/ml of picloram	4 ml	20 ml
ddH_2_O	Volume to 1 L	Volume to 5 L
pH original	4.0	4.0
pH adjusted	5.7	5.7

**Table 4:** The formula of 2× regeneration medium.

Components	1 L	5 L
10**×** L7 macrosalts	200 ml	1,000 ml
1,000**×** L microsalts	2 ml	10 ml
100**×** MS FeNaEDTA	20 ml	100 ml
200**×** vitamins/inositol L	10 ml	50 ml
Maltose	60 g	300 g
1 mg/ml of 2,4-D	0.2 ml	1 ml
10 mg/ml of zeatin	1 ml	5 ml
pH original	4.0	4.0
pH adjusted	5.7	5.7

**Table 5:** The formula of 2× selection medium (Note 6).

Components	1 L	2 L
10**×** L7 macrosalts	200 ml	400 ml
1,000**×** L microsalts	2 ml	4 ml
100**×** MS FeNaEDTA	20 ml	40 ml
200**×** vitamins/inositol L	10 ml	20 ml
Maltose	60 g	120 g
10 mg/ml of PPT	800 µl	1.6 ml
pH original	4.0	4.0
pH adjusted	5.7	5.7

**Table 6:** The formula of 2× rooting medium.

Components	1 L	2 L
10**×** L7 macrosalts	200 ml	400 ml
1,000**×** L microsalts	2 ml	4 ml
100**×** MS FeNaEDTA	20 ml	40 ml
200**×** vitamins/inositol L	10 ml	20 ml
Maltose	60 g	120 g
pH original	4.0	4.0
pH adjusted	5.7	5.7

#### Reagents for the coating of gold particles with DNA

**(1)** 0.1 M of spermidine. To prepare 1 M of spermidine, first, weigh spermidine in a container and dissolve it in distilled water. Calculate the volume of water required according to the following formula, V = g/M × 1,000 ml (M = 145.25 g/mol). Dissolve fully and dispense each 100-µl aliquot of 1 M of spermidine into a 1.5-ml Eppendorf tube. Take one tube of the above 1 M of spermidine, add 900 µl sterile water, and mix well to dilute to 0.1 M of spermidine. Dispense into 23 µl/0.5 ml and store at −20°C (**Note 7**).
**(2)** 2.5 M CaCl_2_. Weigh 3.76 g of CaCl_2_·2H_2_O. Add 10 ml of distilled water and mix very well/vortex. Use 0.2 µm filter sterilization. After sterilization, place each 55-µl aliquot of CaCl_2_ solution into a 1.5-ml of Eppendorf tube and store at −20°C.
**(3)** 40 mg/ml of gold. Accurately weigh 40 mg of gold powder in a 1.5-ml sterile centrifuge tube. Add 1 ml of 100% ethanol into the tube, sonicate for 2 min (and the ultrasound frequency is 40 kHz), centrifuge for 3 s (1,100 *g*), and remove the supernatant. Repeat the above steps two more times. Add 1 ml of SDW to the gold powder, sonicate for 2 min (and the ultrasound frequency is 40 kHz), centrifuge for 3 s (1,500 *g*), and remove the supernatant. Repeat the above steps three times. Resuspend the gold powder in 1 ml of SDW for every 40 mg of gold. Aliquot the gold suspension into 25 µl amounts, vortexing between each aliquot. Store at −20°C.
**(4)** Plasmid DNA. Extract the plasmid DNA with a mini plasmid extraction kit (TianGen TIANprep mini plasmid kit, TIANGEN BIOTECH Co., Beijing, China). The concentration and purity of the plasmid should be determined, diluted in 5 μg/20 µl aliquots, and stored at −20°C (**Note 8**).

## Methods

### Preparation of immature scutella as the explants for transformation

#### Selection of immature scutella for transformation

**Figure 2:**
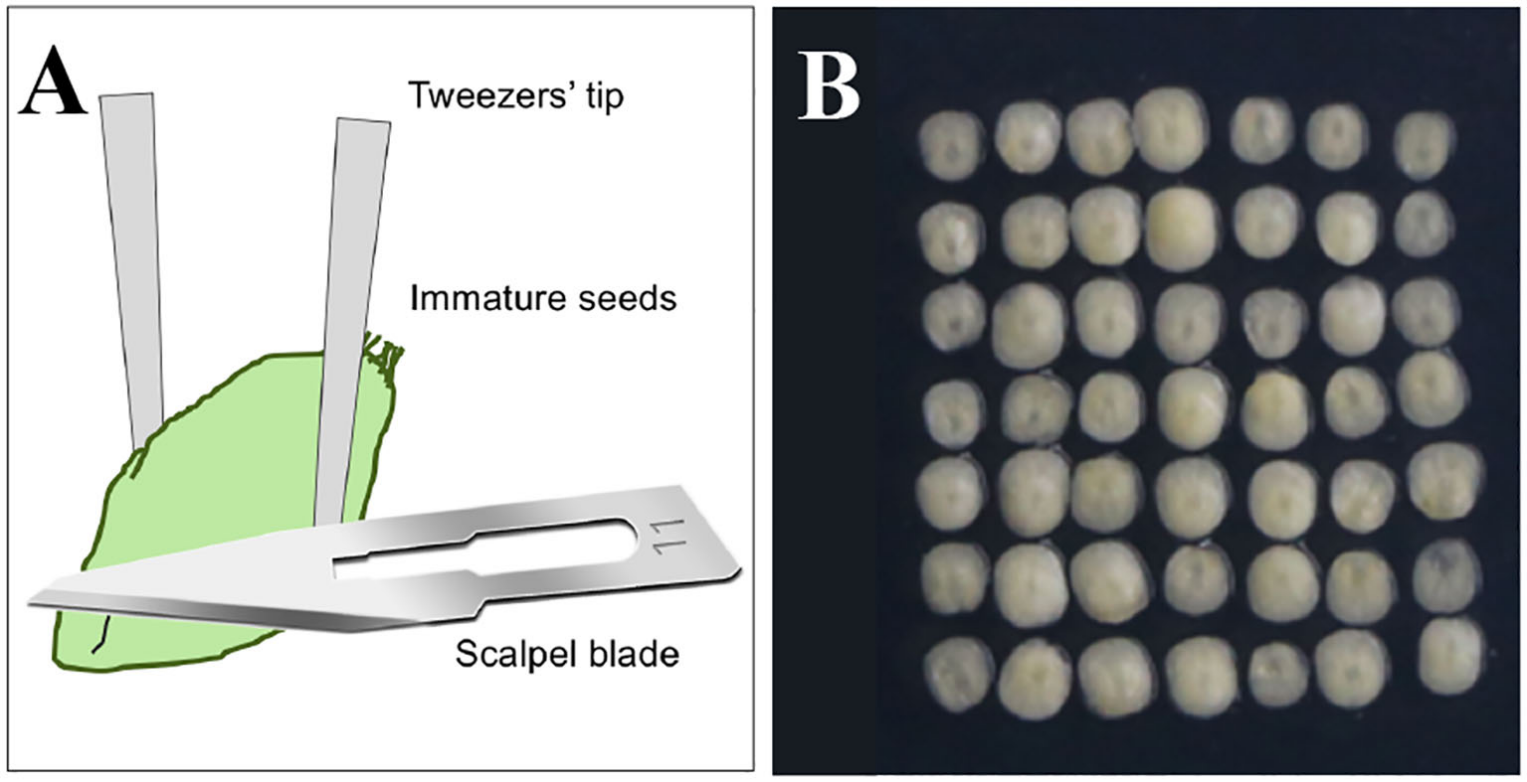
Preparation of the explants. **(A)** The schematic diagram showing details about cutting off the immature embryo axis and isolating the scutella. **(B)** The immature scutella isolated and used for genetic transformation.

**(1)** Collect ears at approximately 12–16 days post-anthesis to isolate immature scutella for wheat transformation (**Note 9**).
**(2)** When the cultivar is 9–10 days post-anthesis, the developmental status of the young embryos in the middle of the spike needs to be observed daily.
**(3) [Critical step]** When the scutellum tissue is translucent, while the embryonic axis is just turning opaque and clearly outlined, with the volume of the young embryo being about 1/2 of the shield, the status of the immature scutella is just right (usually immature embryos reach to this developmental status in about 13–15 days post-anthesis, **Figure 2**). A few seeds can be dissected on-site (either in the field or in the greenhouse) to determine the size and status of the embryos (**Note 10**).
**(4)** When the immature scutella are ready, the spikes with a similar developmental status to the scutella should be collected in a short period, and the collection should avoid daytime with high temperatures (**Note 11**).
**(5)** After collection, the spikes can be kept in water and kept fresh for transformation at 4°C for 24 h.

#### Sterilization of the seeds

**(6)** After spike collection, the disease-free, intact wheat seeds from the middle part of each spike were dissected and placed in a clean 9-cm Petri dish (**Note 12**).
**(7)** In a sterile environment (for example, a laminar flow hood), transfer the wheat seeds into a sterilized conical flask (250 ml), add 75% (v/v) ethanol to submerge the seeds, and shake for 1 min to fully sterilize the seed surface (**Note 13**).
**(8)** After discarding the used 75% ethanol, shake the seeds with SDW two times (3–5 min for each time).
**(9)** Sterilize the seeds with 0.1% HgCl_2_ for 8 min by frequent shaking. After discarding the used HgCl_2_ solution, rinse with SDW three to five times (3–5 min for each time). After HgCl_2_ sterilization, rinse the seeds copiously with at least three changes of water (**Notes 1, 14**).
**(10)** Transfer the seeds to several sterile Petri dishes with lids to maintain the sterilized seeds in moist conditions while without excess water (**Note 15**). The seeds can be used for scutellum isolation immediately or sealed by Parafilm, temporarily stored at 4°C, and taken as they were used (**Note 16**).

#### Isolation of immature scutella and pre-culture

**(11)** Mark the bombardment range at the bottom of a plate of MS induction medium by using the target shelf of the PDS-1000/He particle gun (Bio-Rad, Hercules, CA, USA).
**(12)** In a laminar flow hood, directly isolate and select the immature scutella with the appropriate sizes and suitable conditions by using sterilized tweezers and scalpel blade and remove the embryo axis to prevent precocious germination (the detailed diagram is shown in **Figure 2A**) (**Note 17**).
**(13)** Arrange the wounded side of the excised scutella downward in the bombardment range according to 7 × 7/dish (**Figure 2B**) (**Note 18**).
**(14)** The prepared immature scutella were pre-cultured on the MS induction medium in the dark at 26°C for about 12 h and then used as explants for particle bombardment (**Note 19**).

#### Coating of gold particles with DNA

The following procedure should be carried out on the ice in a sterile environment.

**Figure 3:**
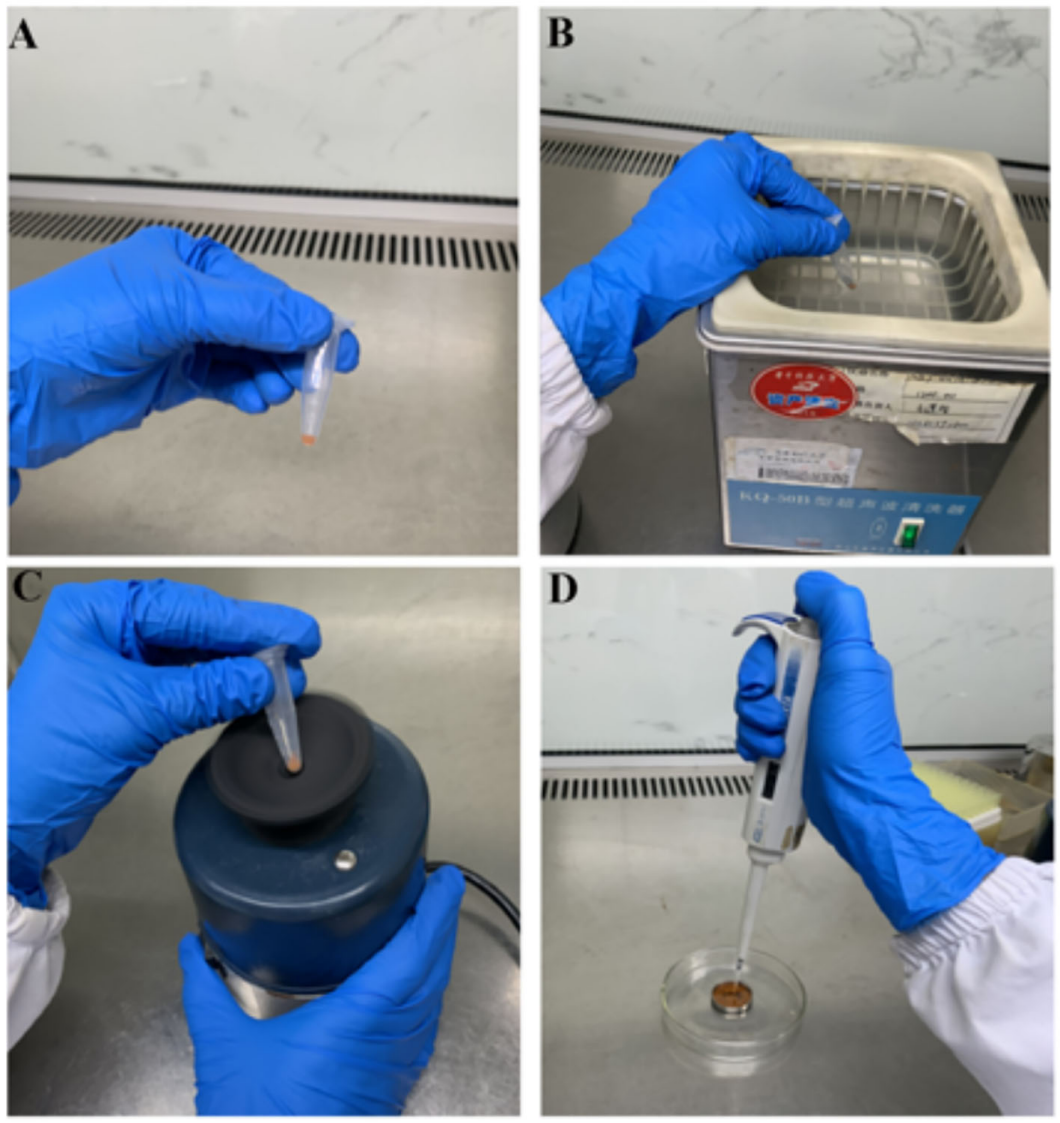
Coating of gold particles with DNA for bombardment. **(A)** Gold powder suspension. **(B)** Ultrasonic treatment for gold powder suspension. **(C)** Vortex treatment for gold powder suspension. **(D)** Plasmid wrapped with gold powder was added to the center of the macrocarrier.

**(15)** After sterilizing the bench top of a laminar flow hood with 75% (v/v) ethanol, sterilize the autoclaved macrocarrier and macrocarrier holder again with 100% ethanol for 1 min and then dry on a sterile stopping screen. The macrocarrier, macrocarrier holder, and the pre-autoclaved stopping screen are left in the hood with the fan and UV light turned on for another 30 min to fully sterilize.
**(16)** Dilute the plasmids with SDW to 5 μg of DNA in 20 μl solution. Meanwhile, place 20 μl of 0.1 mol/L spermidine and 50 μl of 0.1 mol/L CaCl_2_ solution (see section “Reagents for the coating of gold particles with DNA”) on ice (**Note 20**).
**(17)** [Critical step] Take 25 μl of 40 mg/ml suspension of gold powder, ultrasonicate for 1 min (the ultrasound frequency is 40 kHz) followed by vortex for 1 min to make the gold powder fully suspended, and place on ice (**Figure 3A**) (**Note 21**).
**(18)** [Critical step] Add 20 μl of diluted plasmid solution to the wall of the Eppendorf tube of gold-powder suspension. Add 50 μl of the CaCl_2_ solution and 25 μl of the spermidine solution to the cap of the tube of gold-powder suspension and pipette to mix them quickly, snap on the cap, stand the tube upright so that the CaCl_2_–spermidine mixture, and diluted plasmid falls into the gold suspension simultaneously. Then vortex the tube briefly for 5 s and leave the tube at room temperature for 3–5 min (**Figures 3B, C**) (**Note 22**).
**(19)** Place the macrocarrier holder in a sterilized 9-cm Petri dish, and then place the macrocarrier in the macrocarrier holder and flatten it. Repeat this step and assemble seven macrocarrier holders.
**(20)** Take the tube of DNA–gold mixture, centrifuge it at 12,000 rpm for 3 s, and then pipette to get rid of the supernatant.
**(21)** Add 150 μl 100% ethanol to the Eppendorf tube, use the tip of the pipette to scrape the gold particles off the wall, repeatedly aspirate and beat them to make a uniform suspension, and vortex them briefly for 5 s.
**(22)** Repeat step 21.
**(23)** Add 50–60 μl of 100% ethanol to the Eppendorf tube, immediately vortex for 5 s, aspirate 7 µl in its middle, and quickly transfer to the center of the macrocarrier carefully, which has been placed in the macrocarrier holder in Step 15. The 7-µl droplet of DNA-coated gold particles spreads evenly on the macrocarrier. The subsequent six operations were identical to the first. Close the lid of the Petri dish and seal it with Parafilm (**Figure 3D**) (**Note 23**).

#### Particle bombardment

**Figure 4:**
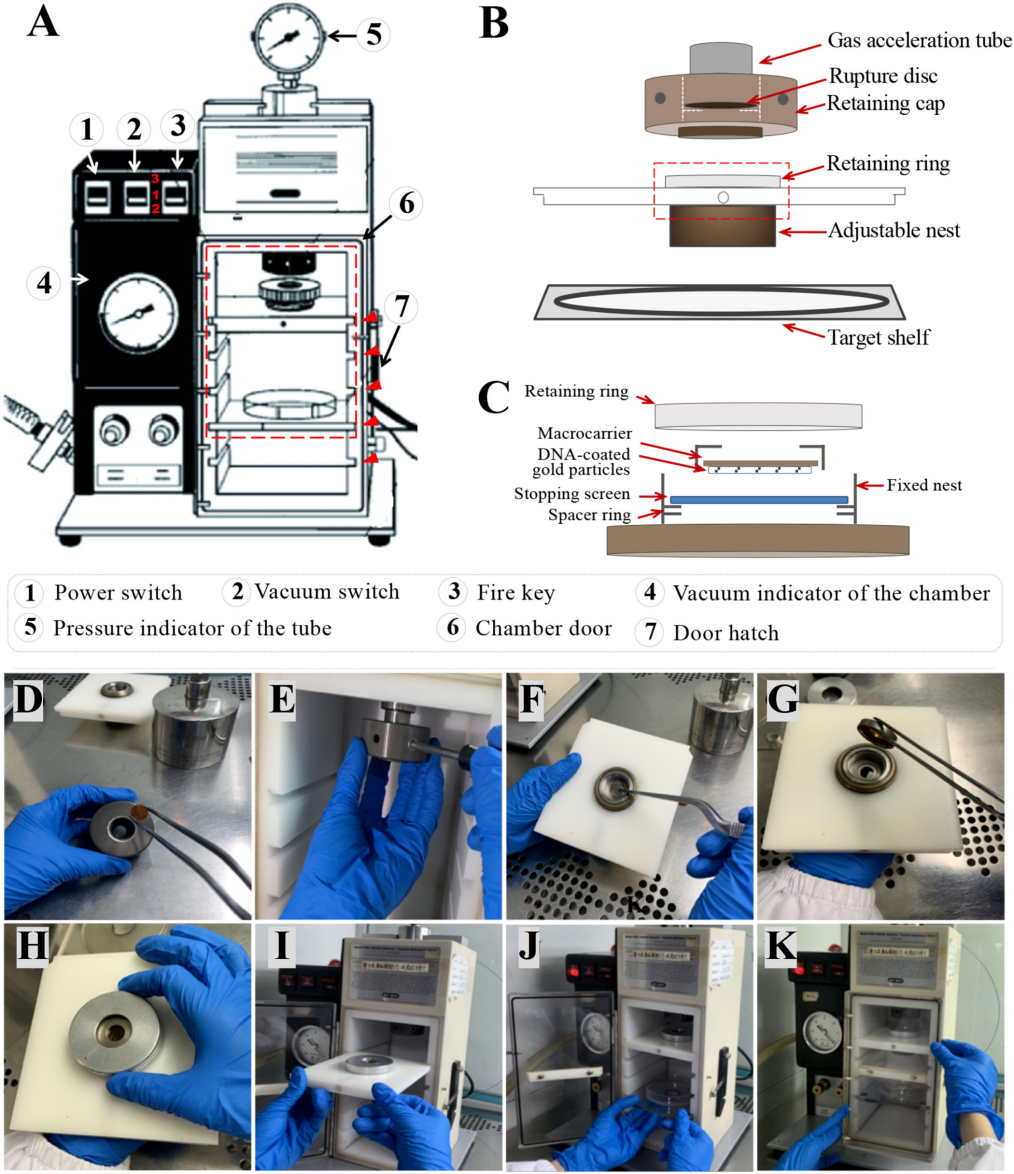
The diagrams of the particle gun, the macrocarrier launch assembly, and the procedures for particle bombardment. **(A)** A photo annotation of the particle gun with each switch and part annotated in the lower panel. The three positions of the vacuum control switch are labeled in red number. The slots for the macrocarrier and the target are indicated with red arrowheads, from top to bottom, being slot 1 to slot 5. **(B)** A schematic diagram illustrating the installation of the accessories into the interior of the particle gun with the details highlighted by the red box shown in panel **(C)**. **(C)** Details of the assembly of macrocarrier. **(D)** The dried rupture disc is put into the rupture disc fixing cap. **(E)** The fixing cap is screwed to the right of the outlet tube with a wrench. **(F)** The dried stopping screen is put into the fixed nest. **(G)** The macrocarrier holder is placed in the fixed nest with the plasmid side turned down. **(H)** The fixing ring is screwed and fixed. **(I)** The macrocarrier launch assembly is loaded into slot 1 of the chamber. **(J)** The destination tray containing the Petri dish is placed in slot 3 of the chamber. **(K)** Close the chamber door.

**(24)** In any bombardment experiment, controls should be included to monitor regeneration and selection efficiencies. The following settings are maintained as standard parameters for particle bombardment: gap 2.5 cm (distance between rupture disc and macrocarrier), stopping plate aperture 0.8 cm (distance between macrocarrier and stopping screen), target distance 5.5 cm (distance between stopping screen and target plate), vacuum 91.4–94.8 kPa, vacuum flow rate 5.0, and vent flow rate 4.5 (**Notes 24, 25**).
**(25)** The PDS-1000/He particle gun (Bio-Rad) is the delivery system for this protocol (**Note 26**), which involves the use of high pressure to accelerate particles to high velocity. Appropriate safety precautions and safety procedures should be taken when operating the instrument.
**(26)** Clean the laminar flow hood where the particle gun is placed with 75% ethanol. Carefully wipe the exterior and interior of the particle gun, the macrocarrier launch assembly, the rupture disc retaining cap, the target disk holder, and related gene gun accessories with 75% ethanol for disinfection (**Figure 3A**).
**(27)** For sterilization purposes, submerge the rupture discs and stopping screens into 100% ethanol for 5 min and allow the aqueous ethanol to evaporate completely on a mesh rack. The rupture discs and stopping screens are left in the hood with the fan and UV light turned on for another 30 min to fully sterilize (**Note 27**).
**(28)** Open the main helium cylinder valve counterclockwise and adjust the secondary valve to the proper pressure, typically 200 psi higher than the rupture disc used.
**(29)** Place an ethanol-dried rupture disc in the rupture disc retaining cap and tighten it to the gas acceleration tube with a wrench to the right, being careful not to tighten it too much. The details of the interior chamber of the particle gun are shown in **Figures 4A–C**.
**(30)** Put an ethanol-dried stopping screen into the fixed nest on the macrocarrier launch assembly, then place the macrocarrier holder with the plasmids facing downwards on it, fix it with the retaining ring, and finally put the macrocarrier launch assembly into slot 1 of the chamber (**Figures 4D–I**).
**(31)** Place the target shelf in slot 3 of the chamber. Place the Petri dish on the target shelf where the immature scutella are pre-cultured on the medium, remove the Petri dish lid, gently push the target stage into the chamber, and close the hatch (**Figures 4J, K**).
**(32)** Turn on the power switch of the particle gun. Turn on the vacuum pump and push the gun’s vacuum switch to position 2 to start to vacuum (**Figure 4A**).
**(33)** When the vacuum indicator turns 27 (No. 4 in **Figure 4A**), push the gun’s vacuum switch to position 3 to hold the vacuum, turn off the pump, and immediately press and hold the fire key until it bursts. After bombardment, release the fire key immediately and return the vacuum switch to position 1 (**Note 28**). While the rupture disc is being burst, note the pressure indicator to check whether the rupture disc is bursting at the expected pressure. This is an important step to ensure a successful shot as if the rupture disc is burst at a higher or lower pressure, the transformation efficiencies may be affected (**Note 29**).
**(34)** When the vacuum indicator needle is zeroed, open the hatch, take out the Petri dish, and put the lid back on.
**(35)** Remove the macrocarrier launch assembly. Unscrew the retaining ring, remove the macrocarrier holder, and place it in 100% ethanol to re-sterilize. Remove the macrocarrier that has been released from the holder and discard it. Remove the stopping screen and place it in 100% ethanol to re-sterilize. Loosen the rupture disc retaining cap, and then unscrew it fully by hand. Remove the burst rupture disc and discard. Repeat the assembly/disassembly process for further shots (**Note 30**).
**(36)** Place the macrocarrier holder and stopping screen in 100% ethanol to re-sterilize if they are to be reused for further shots. Sonicate for 10 min prior to reuse.
**(37)** Last, turn off the pump and the particle gun switch. Turn off the main valve of the helium cylinder clockwise.
**(38)** Sterilize the particle gun’s chamber and its accessories with 75% ethanol, and sterilize the bench top of the hood.

### Tissue culture to regenerate transgenic plants of wheat

#### Induction of embryogenic callus

**Figure 5:**
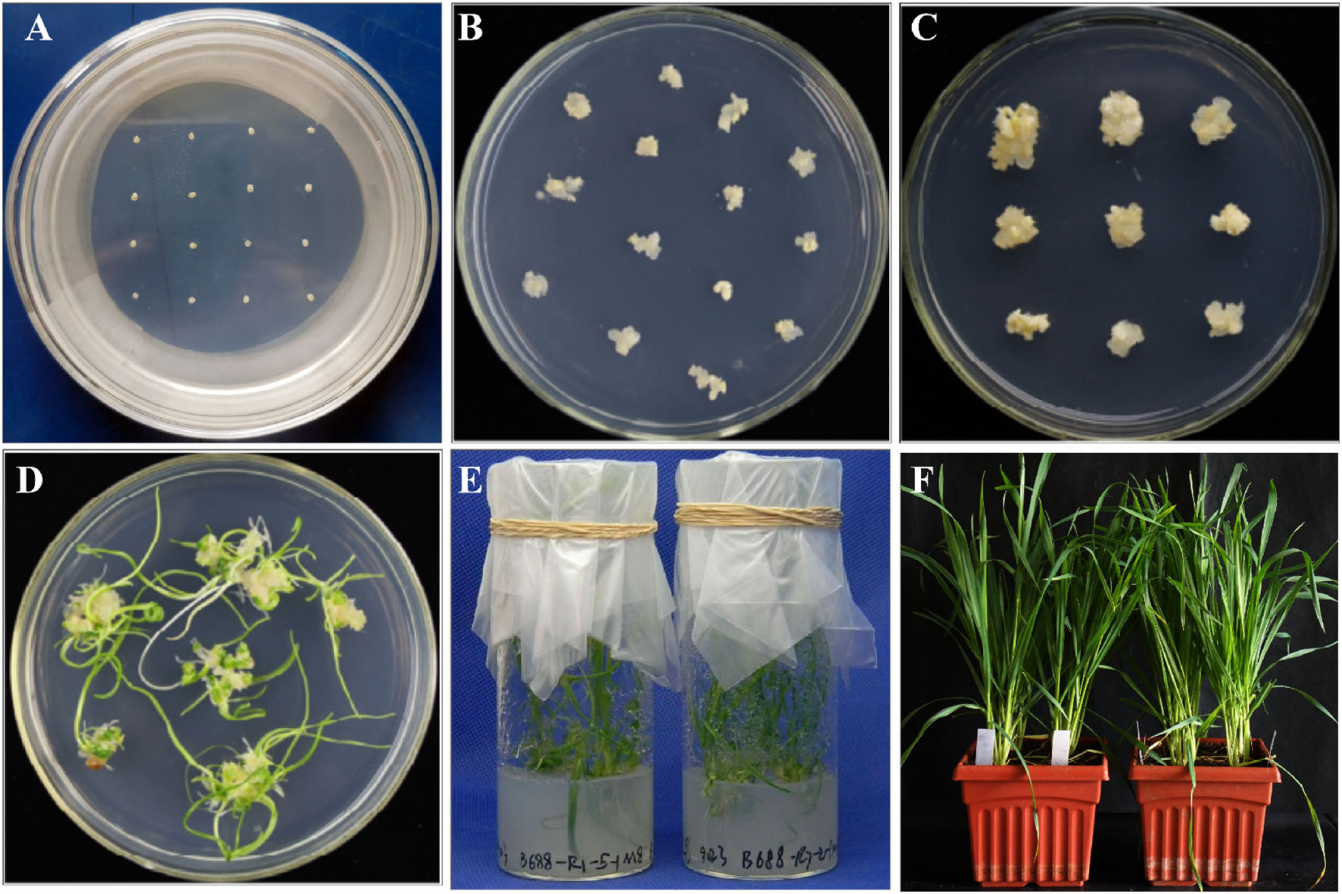
Tissue culture and plant regeneration. **(A)** The immature scutella after particle bombardment. **(B)** Formation of callus from the immature scutella after particle bombardment. **(C)** Enlarged embryogenic callus generated after 3 weeks of induction. **(D)** After 3 weeks’ regeneration, seedlings are grown from the calli. **(E)** Regenerated shoots are transferred to the rooting medium. **(F)** Regenerated plants are transferred to pots for growth in climate chambers.

Following bombardment, the immature scutella were incubated in the dark for 12–24 h and then transferred to new induction medium plates in a sterile environment. Put the scutella more evenly across the medium (approximately 16 per Petri dish). The Petri dishes are quickly sealed with Parafilm and stored in dark for induction culture for about 3 weeks at 22°C (**Figure 5A**) (**Note 31**).

#### Regeneration and selection

After the induction culture, the calli bearing somatic embryogenesis are transferred to the plate of differentiation medium (**Note 22**). The whole calli should be transferred without division, placing approximately 10 calli per plate (**Figure 5**). Incubate at 22°C under light for about 3 to 4 weeks. The photoperiod of the controlled environment is set to 16 h of light and provided by white light lamps. Notably, in the different steps of callus induction, regeneration, and selection, the sub-culture of the callus can be performed (Ismagul et al., 2018; Debernardi et al., 2020).

There are two alternate routes of tissue culture (**Figure 1**): in Route 1, the selection at the rooting stage is performed. Culture the embryogenic callus on the differentiation medium without selection for 3–4 weeks, and transfer all of the calli to the rooting medium with the selection (4 mg/L PPT) only performed in the rooting stage. In Route 2, perform selection first in the regeneration stage. Culture the embryogenic callus on the differentiation medium with selection for 3–4 weeks (4 mg/L PPT), and then transfer the calli that survive on the selection medium to the rooting medium; for Route 2, the selection may or may not be performed at the rooting stage (**Note 33**). In the following, we describe Route 1 in detail to enhance the readability of the protocol.

##### Route 1

After the first 3–4-week regeneration, transfer the calli carefully (without dividing the callus into pieces) to the selection medium plates and incubate at 22°C in the light for another 3–4 weeks (**Note 34**). The selection reagent varies depending on the selectable marker gene used in the plasmid. In our case, *bar* gene is the selectable marker gene, and PPT is the selection reagent.

#### Rooting culture

After the regeneration culture, the differentiated seedlings are transferred to the rooting medium plates to promote root growth (**Note 35**). After approximately 1 month of incubation at 22°C under light conditions, the seedlings became erect and robust, with their root systems developed (**Figures 5E, F**).

#### Transplantation of the seedlings

The rooted wheat seedlings are transferred to nutrient soil, labeled, and kept in the shade for 2 days (23°C–24°C) before planting in a regular greenhouse or growth chamber (23°C–24°C, with a photoperiod of 16 h of the day and 8 h of the night). After the regenerated plants are established in a greenhouse (at least three to four fully expanded leaves), the leaf samples of the regenerated plants are collected for molecular identification of the transgenic-positive plants (**Notes 36, 37**).

### PCR-based identification of transgenic-positive plants

In this protocol, we describe the procedures for identifying transgenic-positive plants by polymerase chain reaction (PCR) analysis, while there are multiple approaches for detecting transgenic plants.

The leaf sample (approximately 2 cm in length) can be collected into an Eppendorf tube for genomic DNA extraction. The tube should be snap-frozen in liquid nitrogen.

Grind leaf samples to powder when it is frozen (**Note 36**); DNA extraction follows the standard cetyltrimethylammonium bromide (CTAB) method.

Design suitable primers for PCR detection (**Note 37**). After the PCR, the amplification results are detected by 1% agarose gel electrophoresis. The specifically amplified PCR band with the correct size is recommended to be purified and to validate the PCR products by Sanger sequencing.

## Results

### Generating transgenic wheat independent of genotypes

After particle bombardment, the immature scutella enlarge on the induction medium and further develop into an embryogenic callus (**Figures 5A, B**). After 3–4 weeks of induction, the majority of explants form embryogenic calli ready to be transferred to the regeneration medium. After regeneration and/or selection, calli differentiate into tentacle-like green shoots with multiple green narrow leaves grown later (**Figures 5C, D**). If the developing callus tissues are moved to the regeneration medium with selection reagents (PPT in this protocol), non-transgenic shoot growth will be substantially inhibited. Similarly, after moving the shoot-grown callus tissues to the rooting medium with selection reagents, non-transgenic root development will be inhibited, thus screening for transgenic events with both shoot and root growth (**Figures 5E, F**). After 1 month in the rooting medium, the putative transgenic callus tissues with the shoot and root growth can be moved to a cold room for vernalization for 2–3 weeks if necessary. With the use of this protocol, established transgenic T_0_ plants can be obtained about 90 days after particle bombardment (**Figure 1**). It generally takes additional approximately 3.5 months in the greenhouse to produce mature T_1_ seeds.

Here, we compile the transgenic lines of wheat generated in the previous studies by our lab (**Table 7**). Transgenic wheat has been successfully produced in a total of 13 genotypes, including six durum wheat cultivars (i.e., L35, Ofanto, Svevo, Luna, Venusia, and Latino X Lira) and seven common wheat genotypes. Among the seven genotypes of common wheat, three are the model cultivars for wheat genetics or flour quality studies (Chinese Spring, Bobwhite, and L88-31) (Lawrence et al., 1988; International Wheat Genome Sequencing Consortium (IWGSC), 2018), and two are the modern elite varieties widely in China (EM12, ZM9023) (Wang et al., 2010; Ma et al., 2013b), with the remaining two lines being Chinese landraces (Baishuimai and Baimangmai). The transformation efficiency was comparable between durum wheat and common wheat, with those ranging from 0.3% to 0.7% in the durum wheat lines (He et al., 1999; Luo et al., 2008). In most of the cases, the transformation efficiency was between about 0.2% and 1.0% for the common wheat genotypes. Notably, transformation using linear expression cassettes tends to achieve higher efficiency than using the plasmids with the same variety and the same transformation parameters (Yao et al., 2006; Yao et al., 2007). Importantly, transformation can be achieved in elite varieties and landraces, which not only simply expand the range of donor genotypes but are useful in genetic improvement and molecular breeding, allowing validating gene functions in particular genetic backgrounds or stacking genes of interest in a major, elite variety. It is also worth noting that the majority of the listed transformation experiments aim at gene functional studies and these transgenic lines were created by different students, rather than solely purposed for optimization of the transformation system for a certain genotype. Different operators for generating transgenic wheat lines may probably differ in the proficiency of lab skills, such as sterile techniques, proper sampling of immature seeds, and scutellum isolation. Thus, the transgenic efficiencies for the same cultivar have varied between studies. However, this well demonstrates that the method described here is robust for different wheat genotypes and different operational persons.

**Table 7:** Summary of the selected transgenic wheat lines produced by our lab.

The plasmids containinggenes of interest	The plasmids containing the marker genes	Selectablemarker	Visiblemarker	Cultivar	No. of explants	No. of positive events	Trans-formation efficiency(%)	References
A. p1Dx5**^a^** B. p1Ax1	A. pDE4 + pDE110 B. pAHC25	*bar*	*uidA*	***L35* ^b^**	731	5	0.7	He et al., 1999
A. p1Dx5 B. p1Ax1	A. pDE4 + pDE110 B. pAHC25	*bar*	*uidA*	***Ofanto***	331	2	0.6	He et al., 1999
A. p1Dx5 B. p1Ax1	A. pDE4 + pDE110 B. pAHC25	*bar*	*uidA*	***Svevo***	327	2	0.6	He et al., 1999
A. p1Dx5 B. p1Ax1	A. pDE4 + pDE110 B. pAHC25	*bar*	*uidA*	***L X L***	294	1	0.3	He et al., 1999
–	pAHC25	*bar*	*uidA*	EM12	995	2	0.2	Yao et al., 2006
–	gus + bar E.C.	*bar*	*uidA*	EM12	993	5	0.5	Yao et al., 2006
p1Ax1	pAHC20	*bar*	–	EM12	998	3	0.3	Yao et al., 2006
1Ax1 E.C.	bar E.C.	*bar*	–	EM12	1,010	6	0.59	Yao et al., 2006
–	pAHC25	*bar*	*uidA*	EM12	1,010	4	0.4	Yao et al., 2007
–	gus + bar E.C.	*bar*	*uidA*	EM12	1,006	11	1.1	Yao et al., 2007
pUbi-pinA	pCa-bar	*bar*	*uidA*	***Luna***	~1,000**^c^**	7	0.7	Luo et al., 2008
pUbi-pinA	pCa-bar	*bar*	*uidA*	***Venusia***	~1,000	6	0.6	Luo et al., 2008
pLRPT-Y1+pTP-CRTI	pAHC20 pAHC25	*bar*	*uidA*	EM12	~1,200	12	1.0	Cong et al., 2009
pLRPT-avel	pAHC25	*bar*	*uidA*	ZM 9023	~2,700	25	0.93	Ma et al., 2013a
pLRPT-avel	pAHC25	*bar*	*uidA*	EM 12	~2,700	5	0.19	Ma et al., 2013b
pLRPT-CrtB	pAHC20	*bar*	–	Bobwhite	~1,200	5	0.42	Wang et al., 2014
pUC18-CrtI	pAHC20	*bar*	–	Bobwhite	~1,200	7	0.58	Wang et al., 2014
pLRPT-CrtB + pUC18-CrtI	pAHC20	*bar*	–	Bobwhite	~1,200	3	0.25	Wang et al., 2014
pLRPT-HYD	pAHC20	*bar*	–	Bobwhite	~1,200	2	0.17	Zeng et al., 2015a
pLRPT-HYD-RNAi	pAHC20	*bar*	–	Bobwhite	~1,200	6	0.5	Zeng et al., 2015b
pLRPT-TaLCYB-RNAi	–	–	–	CS	~1,200	5	0.42	Zeng et al., 2015b
pMin-TaCIPK 25	–	–	–	CS	~1,029	5	0.49	Jin et al., 2016
pLRPT-Glu-Mut1Ax1	–	–	–	L88-31	~1,029	6	0.58	Li et al., 2017
pLRPT-Glu-WT1Ax1	–	–	–	L88-31	~1,029	2	0.19	Li et al., 2017
pLRPT-WT avenin-like b	–	–	–	ZM 9023	~1,029	5	0.49	Wang et al., 2019
pLRPT-Mut avenin-like b	–	–	–	ZM 9023	~1,029	14	1.36	Wang et al., 2019
pLRPT-DsRED	–	–	–	CS	~1,200	3	0.25	Su et al., 2019 BP
pMD18-T-TaSPL13-2B	–	–	–	Bobwhite	~1,029	7	0.68	Li et al., 2020
pAHC25-TaLWD1L-A	pAHC25	*bar*	*uidA*	Bobwhite	~1,029	6	0.58	Hu et al., 2020
pAHC25-TaASR1-D	pAHC25	*bar*	*uidA*	ZM 9023	~1,029	4	0.39	Qiu et al., 2021
pMD18-T-TaSPL13-2B	pAHC25	*bar*		Baishuimai	686	4	0.58	Li et al., unpublished
pMD18-T-TaSPL13-2B	pAHC25	*bar*		Baimangmai	686	3	0.43	Li et al., unpublished

^a^ For the transgenic plants of durum wheat reported by He et al. (1999), there are two types of transgenic events with different combinations of constructs, while the number of explants and number of positive events were reported in total for each of the donor cultivars.

^b^ The durum wheat cultivars used for transformation are shown in bold and italics.

### Molecular identification of transgenic-positive lines

**Figure 6:**
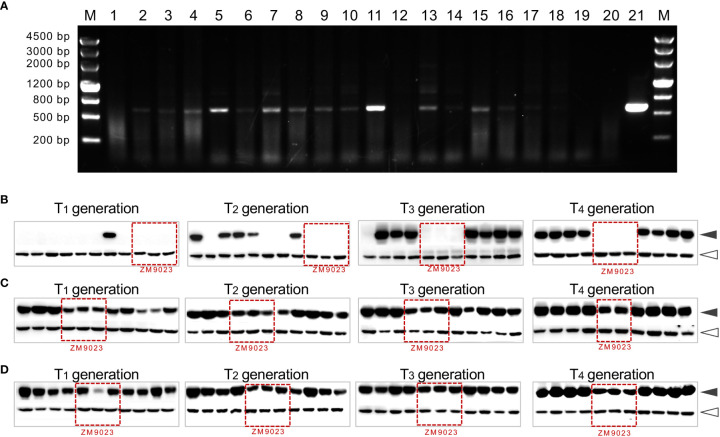
Molecular identification of transgenic-positive plants. **(A)** PCR-based identification of transgenic plants. Lanes 1 to 18 are individual T_1_ seedlings with segregation of the transgene, with transgenic-positive plants having a specific PCR band (524 bp) (the primers used: F-5′ CGCTGAAATCACCAGTCT 3′; R-5′ ACAAGCATTCCCTTAGCG 3′). M, marker; lane 19 is the transgenic donor cultivar ZM9023; lane 20 is the null segregant line; lane 21 is the plasmid used for transformation serving as a positive control. One PCR primer bounds at the CaMV35S terminator, while the other primer bounds at the *TaALPb* gene sequence. **(B–D)** The Western blotting (WB)-based identification of non-segregating transgenic lines of wheat. Three transgenic lines, Mut-ALPb-myc **(B)**, Mut-ALPb **(C)**, and WT-ALPb **(D)**, were propagated using the single seed, and each random eight seeds harvested from a spike were subject to WB analysis for the four consecutive generations (from T_1_ to T_4_). Details regarding seed protein extraction, antibodies, and Western blotting have been described elsewhere (Wang et al., 2019). Seeds of ZM9023 were used as the negative control (the corresponding lanes indicated with red boxes in each blot). Since ZM9023 contains endogenous ALPb proteins, it reacts with the anti-ALPb antibody **(C, D)** but does not react with the anti-myc antibody **(B)**. Black arrowheads indicate the ALPb proteins; white arrowheads indicate the Actin protein, serving as an internal reference for quantifying ALPb protein abundance.

We used a series of transgenic lines as the representative to emphasize that using a combination of approaches, such as PCR, Western blotting, or visible markers, is effective in the identification of the transgenic-positive plants and in screening non-segregant transgenic line (**Figure S2**). The transgenic lines include three events: one expressing the wild-type avenin-like b protein (WT-ALPb) driven by the endosperm-specific *1Dx5* promoter, one expressing a mutant avenin-like b protein (namely, Mut-ALPb with the Y279C mutation to add an extra cysteine residue in the C-terminal of ALPb), and another expressing Mut ALPb fused with the myc tag (namely, Mut-ALPb-myc, Wang et al., 2019) (**Figures S2A–C**). As shown in **Figure 6A**, PCR is an effective and low-cost method to identify the presence of the gene of interest or the marker genes. Also, we emphasize that the Western blotting-based approach serves as a complement to PCR, effectively detecting the expression of transgenic proteins. It is particularly useful if the gene of interest is fused with a tag, allowing the identification of the expressed protein by using commercially available tag antibodies (**Figures 6B–D**; **Figure S2D**).

We also produced the transgenic lines expressing *DsRED* driven by the ubiquitin promoter (**Figure S2D**) (unpublished) to exemplify that florescence-based markers can help to identify transgenic individuals and to detect the segregation of transgenic lines among progenies (discussed below). Indeed, the transgenic seeds were easily distinguished from the null segregant seeds by using a green light source (excitation wavelength of 510–540 nm, LUYOR Co. Ltd., Shanghai, China) with an observation filter of 600 nm (**Figure S3**). In addition, other reporter genes (e.g., GUS or GFP) can be used for transient or stable expression to confirm the expression of the transgene (Yao et al., 2006; Wang et al., 2007; Yao et al., 2007; Ding et al., 2009; Sparks and Doherty, 2020).

## Discussion

Over the last two decades, the bombardment-mediated transformation has been widely used in gene functional studies and genetic improvement of wheat, and these achievements have been reviewed previously (Jones et al., 2005; Li et al., 2012; He et al., 2015; Han et al., 2017; Shrawat and Armstrong, 2018; Hensel, 2020). While the importance of creating transgenic wheat, a limited number of labs are proficient in the transformation, which has been alleviated to some degree until the recent major improvements in *Agrobacterium*-mediated wheat transformation (Richardson et al., 2014; Wang et al., 2016, Wang et al., 2017, Wang et al., 2022). Thus, we intend to provide this detailed protocol, hoping many laboratories can immediately use it for producing transgenic wheat. Previous papers have presented the core of bombardment-mediated wheat transformation and made deep influences on wheat research (Sparks and Jones, 2004; Sparks and Jones, 2009; Jones and Sparks, 2009; Lazzeri and Jones, 2009; Tamás-Nyitrai et al., 2012; Sparks and Jones, 2014; Sparks and Doherty, 2020). As an update and complement of these papers, we present additional new aspects: 1) a summary of the wheat genotypes that can be successfully transformed *via* particle bombardment; 2) the examples and discussion on the approaches to validate transgenic expression, such as Western blotting to detect the proteins or fluorescence-based markers to visualize the transgenic individual; 3) a full set of details and protocol notes. We discuss herein the advantages of this method and its future improvement.

### Comparison between the particle bombardment- and *Agrobacterium*-mediated transformation methods

Particle bombardment- and *Agrobacterium*-mediated transformation approaches are the two major approaches for wheat genetic engineering but have distinct features. Recently, *Agrobacterium*-mediated transformation has been improved dramatically in wheat, with the transformation efficiencies of the PureWheat technology reaching over 32% (Richardson et al., 2014; Ishida et al., 2015). Later, marker-free transgenic plants can be achieved in 15 commercial Chinese wheat varieties, partly unleashing the genotype specificity of *Agrobacterium*-mediated transformation (Wang et al., 2017).

The *Agrobacterium*-mediated methods have higher transformation efficiency than the bombardment-based methods, thus suitable for large-scale production of transgenic wheat and high-throughput gene functional studies using certain genotypes (e.g., Fielder or Kenong 199). Another advantage is the low copy number and simple integration pattern of the foreign genes, allowing rapid selection of homozygous transgenic lines and conventional genetic studies. Theoretically, the transformation efficiency of an *Agrobacterium*-mediated transformation relies on two factors: 1) the susceptibility to *Agrobacterium* infection and 2) the callus induction and regeneration ability. Wheat genotypes differ in the two factors (Wang et al., 2018). Therefore, efficient *Agrobacterium*-based transformation may be likely limited for certain wheat genotypes. Another feature is that the *Agrobacterium*-based method relies on T-DNA, and thus the size of foreign genes is limited.

The particle bombardment-mediated transformation has unique features, allowing it to fit into special niches or application scenarios with lower efficiency. First, it can deliver any form of DNA, RNA, or protein, which not only is favored by transformation but also more importantly could help develop gene-editing technologies (Altpeter et al., 2005; Svitashev et al., 2015; Gil-Humanes et al., 2017; Liang et al., 2017). Moreover, the method is flexible in the types of DNA to be transformed with, has few limitations in making the constructs, and allows co-transformation of multiple constructs, only requiring the constructs to have a selectable marker gene (**Figure S1**). When using linear DNA fragments, the transformation efficiency could be higher than that of using intact plasmids, and the integration of linear DNA fragments is relatively simple (Yao et al., 2007; Gadaleta et al., 2008; Zhang et al., 2015). Co-transformation of multiple constructs or linear DNA fragments allows the manipulation of several genes of interest.

Second, the transgenic efficiency is theoretically only constrained by the callus induction and regeneration ability. This may explain why the particle bombardment-based transformation tends to show less genotype dependency in wheat. Third, the method leads to transgenic events with a high copy number of the foreign genes. The copies are often located on the same locus, allowing for easy segregation in future generations (Choi et al., 2002).

### The particle bombardment-mediated transformation is compatible with multiple genotypes

In addition to the wheat transformation performed by our lab (**Table 7**), we summarize many recent studies, adding another 17 wheat genotypes transformable by the particle bombardment-based method (**Table S9**). These wheat genotypes include 12 Chinese cultivars, four Western cultivars, and one Japanese cultivar. Moreover, numerous European wheat cultivars have been reported to be transformable by particle-bombardment methods (reviewed by Sherawat et al., 2018). Collectively, almost a hundred genotypes can be readily transformed (Li et al., 2012; He et al., 2015; Shrawat and Armstrong, 2018; Hensel, 2020). The reports from our lab and other groups support that the particle bombardment-mediated transformation is compatible with numerous wheat genotypes to produce transgenic plants.

The significance of wide genotype compatibility for a transformation method may be overlooked previously due to the lack of crop pan-genome knowledge. Crop species with large, complex genomes have substantial intra-species variations, with many genes existing in a few accessions but not in the other accessions (namely, the dispensable genes), not to mention numerous haplotypes associated with functional differences. For example, the rice pan-genome study identifies that approximately 38% of the coding genes are dispensable (Zhao et al., 2018). Similarly, approximately 30%–40% of the genes were classified as dispensable or specific to a single genome in maize and *Brassica napus* (Song et al., 2020; Hufford et al., 2021). In wheat, a comparative analysis of multiple genome assemblies identifies gene-number variation of the high-confidence gene models from Chinese Spring (Walkowiak et al., 2020). A recent pan-genomic analysis of the *Aegilops* species indicates that the probable core gene sets of *Triticum*/*Aegilops* species account for approximately 51.2% of all the gene families (Li et al., 2022). In addition, structural variations between accessions are widely distributed in the genome, some profoundly influencing gene expression (Qin et al., 2021). Such extensive intra-species differences in the gene content and structures as well as variants in the regulatory spaces would impact the genotypic effects of genetic engineering. For example, CRISPR/Cas-based and T-DNA-mutagenized knockout lines of the same *OsSPL* member demonstrate different phenotypes in plant development (Jiang et al., 2020). This suggests that it would be ideal to produce transgenic wheat in the target genotype/cultivar. Gene manipulation in the target genotype may save time for the assessment of the effects of such genetic engineering and molecular breeding for commercial purposes.

### Improvements in the particle bombardment-mediated transformation of wheat

Several factors could explain why particle bombardment-mediated transformation remains challenging, labor-intensive, or time-consuming: 1) long time for the tissue culture process; 2) low transformation efficiency likely due to low callus induction and/or regeneration ability with current tissue culture protocol; 3) longer time to obtain non-segregant transgenic lines, likely due to high copy number and complex insertion patterns of the foreign gene; 4) lack of the application of efficient selection methods to visually and accurately identify transgenic events.

Recent efforts are promising in solving these technical bottlenecks. The meristematic tissues of wheat mature embryos were used as the recipient material to obtain transgenic plants through particle bombardment-based transformation, omitting some tedious steps such as healing induction and regeneration (Hamada et al., 2017). One of the factors contributing to the success of the particle bombardment-based transformation is the uniformity of the DNA/gold suspension. A new, simplified DNA/gold coating method allows nanogram amounts of the minimal expression cassettes to be successfully transformed with the transformation efficiency improved to 9.9% and more single-copy transgenic events identified (Ismagul et al., 2018). Recently, using dephosphorylation cassettes and decreasing the amount of DNA during particle bombardment were found to be an effective method to produce transgenic plants with simple integration patterns and a high transmission rate of the expressed transgene to the progeny (Tassy et al., 2014). In addition, a new bombardment-based transgenic protocol has been developed, allowing efficient transformation in cultivated emmer and *Triticum timopheevii* accessions (Miroshnichenko et al., 2020).

Importantly, recent studies demonstrate that co-expression of morphogenesis genes during *Agrobacterium*-mediated transformation is an effective approach to increase callus induction and regeneration ability in cereal crops. For example, the expression of *ZmBBM*, *ZmWUS2*, and other regenerative-related genes can improve transformation efficiency and broaden transformable genotypes in maize (Lowe et al., 2016; Hoerster et al., 2020). However, overexpression of *WUS2* and *BBM* negatively affects callus differentiation and root growth (Lowe et al., 2018). More recently, the expression of *GRF4–GIF1* chimeras in wheat leads to highly efficient transformation in two tetraploid wheat varieties (ranging from 27% to 96%) (Desert King and Kronos) and successful transformation in two previously non-transformable wheat varieties (Hahn and Cadenze; Debernardi et al., 2020). Another breakthrough is that the expression of *TaWOX5* during tissue culture dramatically improves transformation efficiency for 31 common wheat varieties (Wang et al., 2022). Expectedly, the expression of these morphogenesis genes will be very likely effective to improve the particle bombardment-mediated transformation.

In addition, the selectable markers genes have been expanded during these years. Traditionally, the selection is based on resistance to antibiotics or herbicides, such as *bar* gene. On the one hand, efforts have been made to replace traditional antibiotic/herbicide resistance genes with more suitable and safer selectable markers (**Table 8**). On the other hand, the use of visible markers is a promising approach. Various fluorescence-based markers have been widely used in the transformation of many plant species, such as green fluorescent protein (GFP), red fluorescent protein (RFP), DsRED, and mCherry. Some of these genes (i.e., eYGFPuv and DsRED2) have limited applications even though they demonstrate clear advantages, for instance, observation by the naked eye (Sun et al., 2018; He et al., 2020). We think that a reporter that can be directly visualized in a real-time manner and with fewer limitations in the shape, size, and location of the target samples will be ideal for screening transgenic-positive calli or seeds without antibiotic selection. This would help to reduce the working load of wheat transformation.

**Table 8:** Current status of the representative selectable marker genes used in wheat transformation.

The selectable gene	Target tissue	Cultivar	Transformation efficiency	Reference
*pmi*	Embryogenic calli	Svevo	1.14%	Gadaleta et al., 2006
*bar*	Embryogenic calli	Svevo	0.99%	Gadaleta et al., 2006
*pmi*	Immature embryos	Durum wheat breeding line (TC)	1.5%	Gadaleta et al., 2008
*MsGSA*	Immature embryos	Varano	1.1%	Giancaspro et al., 2012
*Synechococcus hemL*	Immature embryos	Varano	2.8%	Giancaspro et al., 2012

## Conclusions

We report here the most updated protocol of particle bombardment-mediated wheat transformation augmented with details and experimental suggestions. By compiling our data and summarizing the results by other groups, we prove that the particle bombardment-based method can be performed in numerous wheat genotypes with fewer limitations in the constructs. These features are useful for wheat gene functional studies and/or for certain genetic engineering purposes. Our protocol will help to promote the method for wider applications as a robust and routine technique in the wheat research community.

## Data availability statement

The original contributions presented in the study are included in the article/**Supplementary Materials**. Further inquiries can be directed to the corresponding authors.

## Author contributions

YQW, GH, JC, and YL conceived and designed the project. YQW, JZ, HZ, PS, LL, XX, QZ, YNW, RW, YZ, and BY performed the wheat transformation and identified transgenic lines. YQW and PS built the constructs mentioned in the Results and **Supplementary Figures**. YQW, JZ, GH, JC, and YL analyzed the data and drafted the manuscript. All of the authors discussed and revised the manuscript

## References

[B1] Altpeter F. Baisakh N. Beachy R. Bock R. Capell T. Christou P. . (2005). Particle bombardment and the genetic enhancement of crops: myths and realities. Mol. Breed. 15 (3), 305–327. doi: 10.1007/s11032-004-8001-y

[B2] Avni R. Nave M. Barad O. Baruch K. Twardziok S. O. Gundlach H. . (2017). Wild emmer genome architecture and diversity elucidate wheat evolution and domestication. Science 357, 93–97. doi: 10.1126/science.aan0032 28684525

[B3] Barcelo P. Lazzeri P. (1995). Transformation of cereals by microprojectile bombardment of immature inflorescence and scutellum tissues. Methods Mol. Biol. 49, 113–123. doi: 10.1385/0-89603-321-X:113 8563798

[B4] Barro F. Cannell M. E. Lazzeri P. A. Barcelo P. (1998). The influence of auxins on transformation of wheat and tritordeum and analysis of transgene integration patterns in transformants. Theor. Appl. Genet. 97, 684–695. doi: 10.1007/s001220050944

[B5] Barro F. Martin A. Lazzeri P. A. Barcelo P. (1999). Medium optimization for efficient somatic embryogenesis and plant regeneration from immature inflorescences and immature scutella of elite cultivars of wheat, barley and tritordeum. Euphytica 108, 161–167. doi: 10.1023/A:1003676830857

[B6] Cheng H. Liu J. Wen J. Nie X. J. Xu L. H. Chen N. B. . (2019). Frequent intra- and inter-species introgression shapes the landscape of genetic variation in bread wheat. Genome Biol. 20, 136. doi: 10.1186/s13059-019-1744-x 31300020 PMC6624984

[B7] Chen Y. M. Song W. J. Xie X. M. Wang Z. H. Guan P. F. Peng H. R. . (2020). A collinearity-incorporating homology inference strategy for connecting emerging assemblies in triticeae tribe as a pilot practice in the plant pangenomic era. Mol. Plant 13, 1694–1708. doi: 10.1016/j.molp.2020.09.019 32979565

[B8] Choi H. W. Lemaux P. G. Cho M. J. (2002). Use of fluorescence *in situ* hybridization for gross mapping of transgenes and screening for homozygous plants in transgenic barley (*Hordeum vulgare* l.). Theor. Appl. Genet. 106, 92–100. doi: 10.1007/s00122-002-0997-y 12582875

[B9] Cong L. Wang C. Chen L. Liu H. J. Yang G. X. He G. X. (2009). Expression of phytoene synthase1 and carotene desaturase *crtI* genes result in an increase in the total carotenoids content in transgenic elite wheat (*Triticum aestivum* l.). J. Agric. Food Chem. 57, 8652–8660. doi: 10.1021/jf9012218 19694433

[B10] Debernardi J. M. Tricoli D. M. Ercoli M. F. Hayta S. Ronald P. Palatnik J. F. . (2020). A GRF-GIF chimeric protein improves the regeneration efficiency of transgenic plants. Nat. Biotechnol. 38, 1274–1279. doi: 10.1038/s41587-020-0703-0 33046875 PMC7642171

[B11] Ding H. Li S. Gao J. Wang Y. Yang G. He G. (2009). Optimization of agrobacterium-mediated transformation conditions in mature embryos of elite wheat. Mol. Biol. Rep. 36, 29–36. doi: 10.1007/s11033-007-9148-5 17906943

[B12] Gadaleta A. Giancaspro A. Blechl A. Blanco A. (2006). Phosphomannose isomerase, pmi, as a selectable marker gene for durum wheat transformation. J. Cereal Sci. 43 (1), 31–37. doi: 10.1016/j.jcs.2005.06.004

[B13] Gadaleta A. Giancaspro A. Blechl A. E. Blanco A. (2008). A transgenic durum wheat line that is free of marker genes and expresses 1Dy10. J. Cereal Sci. 48 (2), 439–445. doi: 10.1016/j.jcs.2007.11.005

[B14] Gao C. (2021). Genome engineering for crop improvement and future agriculture. Cell 184 (6), 1621–1635. doi: 10.1016/j.cell.2021.01.005 33581057

[B15] Giancaspro A. Rosellini D. Blanco A. Gadaleta A. . (2012). Gabaculine selection using bacterial and plant marker genes (GSA-AT) in durum wheat transformation. Plant Cell Tiss. Organ Cult. 109 (3), 447–455. doi: 10.1007/s11240-011-0109-2

[B16] Gil-Humanes J. Wang Y. Liang Z. Shan Q. Ozuna C. V. Sánchez-León S. . (2017). High-efficiency gene targeting in hexaploid wheat using DNA replicons and CRISPR/Cas9. Plant J. 89, 1251–1262. doi: 10.1111/tpj.13446 27943461 PMC8439346

[B17] Goodwin J. Pastori G. Davey M. Jones H. D. (2005). Selectable markers: antibiotic and herbicide resistance. Methods Mol. Biol. 286, 191–202. doi: 10.1385/1-59259-827-7:191 15310922

[B18] Hamada H. Linghu Q. Nagira Y. Miki R. Taoka N. Imai R. (2017). An planta biolistic method for stable wheat transformation. Sci. Rep. 7 (1). doi: 10.1038/s41598-017-11936-0 PMC5597576 28904403

[B19] Han J. Yu X. F. Chang J. L. Yang G. X. He G. Y. (2017). Overview of the wheat genetic transformation and breeding status in China. Methods Mol. Biol. 1679, 37–60. doi: 10.1007/978-1-4939-7337-8_3 28913793

[B20] Hazard B. Trafford K. Lovegrove A. Griffiths S. Uauy C. Shewry P. R. (2020). Strategies to improve wheat for human health. Nat. Food 1, 475–480. doi: 10.1038/s43016-020-0134-6 37128081

[B21] He Y. Jones H. D. Chen S. Chen X. M. Wang D. W. Li K. X. . (2010). Agrobacterium-mediated transformation of durum wheat (*Triticum turgidum* l. var. *durum* cv Stewart) with improved efficiency. J. Exp. Bot. 61, 1567–1581. doi: 10.1093/jxb/erq035 20202997 PMC2852660

[B22] He G. Y. Lazzeri P. A. (1998). Analysis and optimisation of DNA delivery into wheat scutellum and tritordeum inflorescence explants by tissue electroporation. Plant Cell Rep. 18, 64–70. doi: 10.1007/s002990050533

[B23] He G. Y. Lazzeri P. A. (2001). Improvement of somatic embryogenesis and plant regeneration from durum wheat (*Triticum turgidum* var. *durum* desf.) scutellum and inflorescence cultures. Euphytica 119, 369–376. doi: 10.1023/A:1017587628995

[B24] He G. Y. Lazzeri P. A. Cannell M. E. (2001). Fertile transgenic plants obtained from tritordeum inflorescences by tissue electroporation. Plant Cell Rep. 20, 67–72. doi: 10.1007/s002990000285 30759915

[B25] Hensel G. (2020). Genetic transformation of triticeae cereals – summary of almost three decade's development. Biotechnol. Adv. 40, 107484. doi: 10.1016/j.biotechadv.2019.107484 31751606

[B26] He G. Y. Rooke L. Steele S. Békés F. Gras P. Tatham A. S. . (1999). Transformation of pasta wheat (*Triticum turgidum* l. var. *durum*) with high-molecular-weight glutenin subunit genes and modification of dough functionality. Mol. Breed. 5, 377–386. doi: 10.1023/A:1009681321708

[B27] He Y. Wang Q. Zeng J. Sun T. Yang G. X. He G. Y. (2015). Current status and trends of wheat genetic transformation studies in China. J. Integr. Agric. 14, 438–452. doi: 10.1016/S2095-3119(14)60934-5

[B28] He Y. B. Zhang T. Sun H. Zhan H. D. Zhao Y. D. (2020). A reporter for noninvasively monitoring gene expression and plant transformation. Hortic. Res. 7, 152. doi: 10.1038/s41438-020-00390-1 33024566 PMC7502077

[B29] Hoerster G. Wang N. Ryan L. Wu E. Anand A. McBride K. . (2020). Use of non-integrating zm-Wus2 vectors to enhance maize transformation. In Vitro Cell Dev. Biol. Plant 56 (3), 265–279. doi: 10.1007/s11627-019-10042-2

[B30] Hufford M. B. Seetharam A. S. Woodhouse M. R. Chougule K. M. Ou S. J. Liu J. N. . (2021). *De novo* assembly, annotation, and comparative analysis of 26 diverse maize genomes. Science 373, 655–662. doi: 10.1126/science.abg5289 34353948 PMC8733867

[B31] Hu R. Xiao J. Zhang Q. Gu T. Chang J. L. Yang G. X. . (2020). A light-regulated gene, *TaLWD1L-a*, affects flowering time in transgenic wheat (*Triticum aestivum* l.). Plant Sci. 299, 11. doi: 10.1016/j.plantsci.2020.110623 32900433

[B32] International Wheat Genome Sequencing Consortium (IWGSC) Appels R. Eversole K. Stein N. Feuillet C. Keller B. . (2018). Shifting the limits in wheat research and breeding using a fully annotated reference genome. Science 361, eaar7191. doi: 10.1126/science.aar719 30115783

[B33] Ishida Y. Tsunashima M. Hiei Y. Komari T. (2015). Wheat (*Triticum aestivum* l.) transformation using immature embryos. Methods Mol. Biol. 1223, 189–198. doi: 10.1007/978-1-4939-1695-5_15 25300841

[B34] Ismagul A. Yang N. Maltseva E. Iskakova G. Mazonka I. Skiba Y. . (2018). A biolistic method for high-throughput production of transgenic wheat plants with single gene insertions. BMC Plant Biol. 18, 135. doi: 10.1186/s12870-018-1326-1 29940859 PMC6020210

[B35] Jiang M. He Y. Chen X. Zhang X. Guo Y. Yang S. . (2020). CRISPR-based assessment of genomic structure in the conserved SQUAMOSA promoter-binding-like gene clusters in rice. Plant J. 104, 1301–1314. doi: 10.1111/tpj.15001 32996244

[B36] Jin X. Sun T. Wang X. T. Su P. P. Ma J. F. He G. Y. . (2016). Wheat CBL-interacting protein kinase 25 negatively regulates salt tolerance in transgenic wheat. Sci. Rep. 6, 28884. doi: 10.1038/srep28884 27358166 PMC4928124

[B37] Jones H. D. Doherty A. Wu H. (2005). Review of methodologies and a protocol for the agrobacterium-mediated transformation of wheat. Plant Methods 1, 5. doi: 10.1186/1746-4811-1-5 16270934 PMC1277018

[B38] Jones H. D. Sparks C. A. (2009). Stable transformation of plants. Methods Mol. Biol. 513, 111–130. doi: 10.1007/978-1-59745-427-8_7 19347645

[B39] Juliana P. Poland J. Huerta-Espino J. Shrestha S. Crossa J. Crespo-Herrera L. . (2019). Improving grain yield, stress resilience and quality of bread wheat using large-scale genomics. Nat. Genet. 51, 1530–1539. doi: 10.1038/s41588-019-0496-6 31548720

[B40] Lawrence G. J. MacRitchie F. Wrigley C. W. (1988). Dough and baking quality of wheat lines deficient in glutenin subunits controlled by the *Glu-A1*, *Glu-B1* and *Glu-D1* loci. J. Cereal Sci. 7, 109–112. doi: 10.1016/S0733-5210(88)80012-2

[B41] Lazzeri P. A. Jones H. D. (2009). Transgenic wheat, barley and oats: Production and characterization. Methods Mol. Biol. 478, 3–20. doi: 10.1007/978-1-59745-379-0_1 19009436

[B42] Liang Z. Chen K. Li T. Zhang Y. Wang Y. Zhao Q. . (2017). Efficient DNA-free genome editing of bread wheat using CRISPR/Cas9 ribonucleoprotein complexes. Nat. Commun. 8, 14261. doi: 10.1038/ncomms14261 28098143 PMC5253684

[B43] Ling H. Q. Ma B. Shi X. L. Liu H. Dong L. L. Sun H. . (2018). Genome sequence of the progenitor of wheat a subgenome *Triticum urartu* . Nature 557, 424–428. doi: 10.1038/s41586-018-0108-0 29743678 PMC6784869

[B44] Li L. Shi F. Wang Y. Q. Yu X. F. Zhi J. J. Guan Y. B. . (2020). *TaSPL13* regulates inflorescence architecture and development in transgenic wheat (*Triticum aestivum* l.). Plant Sci. 296, 110516. doi: 10.1016/j.plantsci.2020.110516 32539997

[B45] Li M. Wang Y. Q. Ma F. Y. Zeng J. Chang J. L. Chen M. J. . (2017). Effect of extra cysteine residue of new mutant 1Ax1 subunit on the functional properties of common wheat. Sci. Rep. 7, 7510. doi: 10.1038/s41598-017-07541-w 28790347 PMC5548925

[B46] Li J. R. Ye X. G. An B. Y. Du L. P. Xu H. J. (2012). Genetic transformation of wheat: current status and future prospects. Plant Biotechnol. Rep. 6, 183–193. doi: 10.1007/s11816-011-0213-0

[B47] Li L. F. Zhang Z. B. Wang Z. H. Li N. Sha Y. Wang X. F. . (2022). Genome sequences of the five sitopsis species of *Aegilops* and the origin of polyploid wheat b-subgenome. Mol. Plant 15, 488–503. doi: 10.1016/j.molp.2021.12.019 34979290

[B48] Liu G. Lin Q. Jin S. Gao C. (2022). The CRISPR-Cas toolbox and gene editing technologies. Mol. Cell 82 (2), 333–347. doi: 10.1016/j.molcel.20 34968414

[B49] Lowe K. La Rota M. Hoerster G. Hastings C. Wang N. Chamberlin M. . (2018). Rapid genotype ‘independent’ zea mays l. (maize) transformation *via* direct somatic embryogenesis. In Vitro Cell Dev. Biol. Plant 54 (3), 240–252. doi: 10.1007/s11627-018-9905-2 29780216 PMC5954046

[B50] Lowe K. Wu E. Wang N. Hoerster G. Hastings C. Cho M.-J. . (2016). Morphogenic regulators *Baby boom* and *Wuschel* improve monocot transformation. Plant Cell 28 (9), 1998–2015. doi: 10.1105/tpc.16.00124 27600536 PMC5059793

[B51] Luo M. C. Gu Y. Q. Puiu D. Wang H. Twardziok S. O. Deal K. R. . (2017). Genome sequence of the progenitor of the wheat d genome *Aegilops tauschii* . Nature 551, 498–502. doi: 10.1038/nature24486 29143815 PMC7416625

[B52] Luo L. T. Zhang J. R. Yang G. X. Li Y. Li K. X. He G. Y. (2008). Expression of puroindoline a enhances leaf rust resistance in transgenic tetraploid wheat. Mol. Biol. Rep. 35, 195–200. doi: 10.1007/s11033-007-9070-x 17380426

[B53] Luo J. Li S. Xu J. Yan L. Ma Y. Xia L. (2021). Pyramiding favorable alleles in an elite wheat variety in one generation by CRISPR-Cas9-mediated multiplex gene editing. Mol. Plant 14 (6), 847–850. doi: 10.1016/j.molp.2021.03.024 33812982

[B54] Maccaferri M. Harris N. S. Twardziok S. O. Pasam R. K. Gundlach H. Spannagl M. . (2019). Durum wheat genome highlights past domestication signatures and future improvement targets. Nat. Genet. 51, 885–895. doi: 10.1038/s41588-019-0381-3 30962619

[B55] Ma F. Y. Li M. Li T. T. Liu W. Li Y. Hu W. . (2013b). Overexpression of avenin-like b proteins in bread wheat (*Triticum aestivum* l.) improves dough mixing properties by their incorporation into glutenin polymers. PloS One 8, e66758. doi: 10.1371/journal.pone.0066758 23843964 PMC3699606

[B56] Ma F. Y. Li M. Yu L. L. Li Y. Liu Y. Y. Li T. T. . (2013a). Transformation of common wheat (*Triticum aestivum* l.) with *avenin-like* b gene improves flour mixing properties. Mol. Breed. 32, 853–865. doi: 10.1007/S11032-013-9913-1 24288453 PMC3830129

[B57] Miroshnichenko D. Klementyeva A. Pushin A. Dolgov S. (2020). A competence of embryo-derived tissues of tetraploid cultivated wheat species *Triticum dicoccum* and *Triticum timopheevii* for efficient and stable transgenesis mediated by particle inflow gun. BMC Plant Biol. 20 (Suppl 1), 442. doi: 10.1186/s12870-020-02580-4 33050908 PMC7557024

[B58] Pang Y. L. Liu C. X. Wang D. F. St. Amand P. Bernardo A. Li W. H. . (2020). High-resolution genome-wide association study identifies genomic regions and candidate genes for important agronomic traits in wheat. Mol. Plant 13, 1311–1327. doi: 10.1016/j.molp.2020.07.008 32702458

[B59] Pastori G. M. Wilkinson M. D. Steele S. H. Sparks C. A. Jones H. D. Parry M. A. J. (2001). Age-dependent transformation frequency in elite wheat varieties. J. Exp. Bot. 52, 857–863. doi: 10.1093/jexbot/52.357.857 11413223

[B60] Pont C. Leory T. Seidel M. Tondelli A. Duchemin W. Armisen D. . (2019). Tracing the ancestry of modern bread wheats. Nat. Genet. 51, 905–911. doi: 10.1038/s41588-019-0393-z 31043760

[B61] Purugganan M. D. Jackson S. A. (2021). Advancing crop genomics from lab to field. Nat. Genet. 53 (5), 595–601. doi: 10.1038/s41588-021-00866-3 33958781

[B62] Qin P. Lu H. W. Du H. L. Wang H. Chen W. L. Chen Z. . (2021). Pan-genome analysis of 33 genetically diverse rice accessions reveals hidden genomic variations. Cell 184, 3542–3558. doi: 10.1016/j.cell.2021.04.046 34051138

[B63] Qiu D. Hu W. Zhou Y. Xiao J. Hu R. Wei Q. H. . (2021). *TaASR1-d* confers abiotic stress resistance by affecting ROS accumulation and ABA signalling in transgenic wheat. Plant Biotechnol. J. 19, 1588–1601. doi: 10.1111/pbi.13572 33638922 PMC8384601

[B64] Ramírez-González R. H. Borrill P. Lang D. Harrington S. A. Brinton J. Venturini L. . (2018). The transcriptional landscape of polyploid wheat. Science 361, eaar6089. doi: 10.1126/science.aar6089 30115782

[B65] Rasco-Gaunt S. Riley A. Barcelo P. Lazzeri P. A. (1999). Analysis of particle bombardment parameters to optimise DNA delivery into wheat tissues. Plant Cell Rep. 19, 118–127. doi: 10.1007/s002990050721 30754736

[B66] Rasco-Gaunt S. Riley A. Cannell M. Barcelo P. Lazzeri P. A. (2001). Procedures allowing the transformation of a range of European elite wheat (*Triticum aestivum* l.) varieties *via* particle bombardment. J. Exp. Bot. 52, 865–874. doi: 10.1093/jexbot/52.357.865 11413224

[B67] Richardson T. Thistleton J. Higgins T. J. Howitt C. Ayliffe M. (2014). Efficient agrobacterium transformation of elite wheat germplasm without selection. Plant Cell Tiss. Organ. Cult. 119, 647–659. doi: 10.1007/s11240-014-0564-7

[B68] Sansaloni C. Franco J. Santos B. Percival-Alwyn L. Singh S. Petroli C. . (2020). Diversity analysis of 80,000 wheat accessions reveals consequences and opportunities of selection footprints. Nat. Commun. 11, 4572. doi: 10.1038/s41467-020-18404-w 32917907 PMC7486412

[B69] Shrawat A. K. Armstrong C. L. (2018). Development and application of genetic engineering for wheat improvement. Crit. Rev. Plant Sci. 37, 335–421. doi: 10.1080/07352689.2018.1514718

[B70] Shrawat A. K. Loerz H. (2006). Agrobacterium-mediated transformation of cereals: a promising approach crossing barriers. Plant Biotechnol. J. 4, 575–603. doi: 10.1111/j.1467-7652.2006.00209.x 17309731

[B71] Song J. M. Guan Z. L. Hu J. L. Guo C. C. Yang Z. Q. Wang S. . (2020). Eight high-quality genomes reveal pan-genome architecture and ecotype differentiation of *Brassica napus* . Nat. Plants 6, 34–45. doi: 10.1038/s41477-019-0577-7 31932676 PMC6965005

[B72] Sparks C. A. Jones H. D. (2004). “Transformation of wheat by biolistics,” in Transgenic crops of the world – essential protocols. Ed. Curtis I. S. (Dordrecht: Springer), 19–35.

[B73] Sparks C. A. Jones H. D. (2009). Biolistics transformation of wheat. Methods Mol. Biol. 478, 71–92. doi: 10.1007/978-1-59745-379-0_4 19009439

[B74] Sparks C. A. Jones H. D. (2014). Genetic transformation of wheat *via* particle bombardment. Methods Mol. Biol. 1099, 201–218. doi: 10.1007/978-1-62703-715-0_17 24243206

[B75] Sparks C. A. Doherty A. (2020). Genetic Transformation of Common Wheat (Triticum aestivum L.) Using Biolistics. Methods Mol Biol. 2124, 229–250. doi: 10.1007/978-1-0716-0356-7_12 32277457

[B76] Su P. P. (2019). Isolation, functional analysis and application of tissue-specific promoters in wheat. [dissertation/doctor’s thesis] (Wuhan: Huazhong University of Science and Technology).

[B77] Sun L. Alariqi M. Zhu Y. Li J. Y. Li Z. L. Wang Q. . (2018). Red fluorescent protein (DsRed2), an ideal reporter for cotton genetic transformation and molecular breeding. Crop J. 6, 366–376. doi: 10.1016/j.cj.2018.05.002

[B78] Svitashev S. Young J. K. Schwartz C. Gao H. Falco S. C. Cigan A. M. (2015). Targeted mutagenesis, precise gene editing, and site-specific gene insertion in maize using Cas9 and guide RNA. Plant Physiol. 169, 931–945. doi: 10.1104/pp.15.00793 26269544 PMC4587463

[B79] Tamás-Nyitrai C. Jones H. D. Tamás L. (2012). Biolistic- and agrobacterium-mediated transformation protocols for wheat. Methods Mol. Biol. 877, 357–384. doi: 10.1007/978-1-61779-818-4_27 22610641

[B80] Tassy C. Partier A. Beckert M. Feuillet C. Barret P. (2014). Biolistic transformation of wheat: increased production of plants with simple insertions and heritable transgene expression. Plant Cell Tiss. Organ Cult. 119 (1), 171–181. doi: 10.1007/s11240-014-0524-2

[B81] Vasil V. Castillo A. M. Fromm M. E. Vasil I. K. (1992). Herbicide resistant fertile transgenic wheat plants obtained by microprojectile bombardment of regenerable embryogenic callus. Nat. Biotechnol. 10, 667–674. doi: 10.1038/nbt0692-667

[B82] Walkowiak S. Gao L. L. Monat C. Haberer G. Kassa M. T. Brinton J. . (2020). Multiple wheat genomes reveal global variation in modern breeding. Nature 588, 277–283. doi: 10.1038/s41586-020-2961-x 33239791 PMC7759465

[B83] Wang K. R. Kang L. Anand A. Lazarovits G. Mysore K. S. (2007). Monitoring in *planta* bacterial infection at both cellular and whole-plant levels using the green fluorescent protein variant GFPuv. New Phytol. 174, 212–223. doi: 10.1111/j.1469-8137.2007.01999.x 17335510

[B84] Wang Y. Q. Li M. Guan Y. B. Li L. Sun F. S. Han J. P. . (2019). Effects of an additional cysteine residue of avenin-like b protein by site-directed mutagenesis on dough properties in wheat (*Triticum aestivum* l.). J. Agric. Food Chem. 67, 8559–8572. doi: 10.1021/acs.jafc.9b02814 31298518

[B85] Wang K. Liu H. Y. Du L. Ye X. G. (2017). Generation of marker-free transgenic hexaploid wheat *via* an agrobacterium-mediated co-transformation strategy in commercial Chinese wheat varieties. Plant Biotechnol. J. 15, 614–623. doi: 10.1111/pbi.12660 27862820 PMC5399001

[B86] Wang Y. Li Y. Zhang L. Gao X. Miao Y. Wang C. . (2010). Expression of the 1Ax1 transgene in an elite Chinese wheat variety and its effect on functional properties. J. Sci. Food Agric. 90, 106–111. doi: 10.1016/S0733-5210(88)80012-2 20355019

[B87] Wang K. Riaz B. Ye X. G. (2018). Wheat genome editing expedited by efficient transformation techniques: Progress and perspectives. Crop J. 6, 22–31. doi: 10.1016/j.cj.2017.09.009

[B88] Wang K. Shi L. Liang X. N. Zhao P. Wang W. X. Liu J. X. . (2022). The gene *TaWOX5* overcomes genotype dependency in wheat genetic transformation. Nat. Plants 8, 110–117. doi: 10.1038/s41477-021-01085-8 35027699

[B89] Wang G. P. Yu X. D. Sun Y. W. Jones H. D. Xia L. Q. (2016). Generation of marker- and/or backbone-free transgenic wheat plants *via* agrobacterium-mediated transformation. Front. Plant Sci. 7. doi: 10.3389/fpls.2016.01324 PMC5030305 27708648

[B90] Wang C. Zeng J. Li Y. Hu W. Chen L. Miao Y. J. . (2014). Enrichment of provitamin a content in wheat (*Triticum aestivum* l.) by introduction of the bacterial carotenoid biosynthetic genes *CrtB* and CrtI. J. Exp. Bot. 9, 2545–2556. doi: 10.1093/jxb/eru138 PMC4036513 24692648

[B91] Yao Q. Cong L. Chang J. L. Li K. X. Yang G. X. He G. Y. (2006). Low copy number gene transfer and stable expression in a commercial wheat cultivar *via* particle bombardment. J. Exp. Bot. 57, 3737–3746. doi: 10.1093/jxb/erl145 17032730

[B92] Yao Q. Cong L. He G. Y. Chang J. L. Li K. X. Yang G. X. (2007). Optimization of wheat co-transformation procedure with gene cassettes resulted in an improvement in transformation frequency. Mol. Biol. Rep. 34, 61–67. doi: 10.1007/s11033-006-9016-8 17195929

[B93] Zeng J. Wang C. Chen X. Zang M. L. Yuan C. H. Wang X. T. . (2015a). The lycopene β-cyclase plays a significant role in provitamin a biosynthesis in wheat endosperm. BMC Plant Biol. 15, 112. doi: 10.1186/s12870-015-0514-5 25943989 PMC4433027

[B94] Zeng J. Wang X. T. Miao Y. J. Wang C. Zang M. L. Chen X. . (2015b). Metabolic engineering of wheat provitamin a by simultaneously overexpressing *CrtB* and silencing carotenoid hydroxylase (*TaHYD*). J. Agric. Food Chem. 63, 9083–9092. doi: 10.1021/acs.jafc.5b04279 26424551

[B95] Zhang K. Liu J. X. Zhang Y. Yang Z. M. Gao C. X. (2015). Biolistic genetic transformation of a wide range of Chinese elite wheat (Triticum aestivum L.) varieties. J. Genet. Genomics 42, 39–42. doi: 10.1016/j.jgg.2014.11.005 25619601

[B96] Zhao Q. Feng Q. Lu H. Y. Li Y. Wang A. H. Tian Q. L. . (2018). Pan-genome analysis highlights the extent of genomic variation in cultivated and wild rice. Nat. Genet. 50, 278–284. doi: 10.1038/s41588-018-0041-z 29335547

[B97] Zhao G. Y. Zou C. Li K. Wang K. Li T. B. Gao L. F. . (2017). The *Aegilops tauschii* genome reveals multiple impacts of transposons. Nat. Plants 3, 946–955. doi: 10.1038/s41477-017-0067-8 29158546

