# Supplementary material for: An established protocol for generating transgenic wheat for wheat functional genomics via particle bombardment
Source: Front Plant Sci. 2022 Dec 8;13:979540. doi: 10.3389/fpls.2022.979540 (PMC9772560; doi:10.3389/fpls.2022.979540)
Supplement: Supplementary file 1 [file DataSheet_1.docx]

Supplementary Information for

An Established Protocol for Generating Transgenic Wheat for Wheat Functional Genomics via Particle Bombardment

Yaqiong Wang^1,#^, Jian Zeng^2,#^, Peipei Su^1,3^, Hongyan Zhao^1^, Li Li^1^, Xiaoxue Xie^1^, Qian Zhang^1^, Ya’nan Wu^1^, Ruibin Wang^1^, Yufan Zhang^1^, Boju Yu^1^, Mingjie Chen^1^, Yuesheng Wang^1^, Guangxiao Yang^1^, Guangyuan He^1,^*, Junli Chang^1,^*, Yin Li^1,^*

^1^The Genetic Engineering International Cooperation Base of Chinese Ministry of Science and Technology, The Key Laboratory of Molecular Biophysics of Chinese Ministry of Education, College of Life Science and Technology, Huazhong University of Science & Technology, Wuhan 430074, China

^2^Guangdong Provincial Key Laboratory of Utilization and Conservation of Food and Medicinal Resources in Northern Region, Shaoguan University, Shaoguan, Guangdong 512005, China

^3^present address: College of Life Science and Resources and Environment, Yichun University, Yichun, Jiangxi Province 336000, China

^#^These authors contributed equally to the work.

***Correspondence:**

Guangyuan He: hegy@hust.edu.cn

Junli Chang: cjl@hust.edu.cn

Yin Li: yinli2021@hust.edu.cn

**This file contains:**

**Table S1 |** The formula of 10×MS Macrosalts stock solution.

**Table S2 |** The formula of 10×L Microsalts stock solution.

**Table S3 |** The formula of 100×MS FeNaETA stock solution.

**Table S4 |** The formula of 1000×MS vitamins (-Glycine) stock solution.

**Table S5 |** The formula of 25×3AA (Amino Acid) stock solution.

**Table S6 |** The formula of 100 mg/mL myo-inositol stock solution.

**Table S7 |** The formula of 10×L7 Macrosalts stock solution.

**Table S8 |** The formula of 200×L vitamins&inositol stock solution.

**Table S9 |** Summary of the reported work of wheat genetic transformation mediated by particle gun.

**Figure S1 |** Diagram of the three representative vectors used for particle bombardment-mediated wheat transformation.

**Figure S2 |** The diagram of the constructs used for generating transgenic wheat lines is shown in Figure 6 and Figure S3.

**Figure S3 |** Identification of transgenic wheat seeds expressing the visible marker DsRED by fluorescent detection with the naked eye.

**Supplementary Notes**

**Supplementary References**

**Table S1 |** The formula of 10×MS Macrosalts stock solution.

| **Components** | **1 L** | **5 L** |
| --- | --- | --- |
| NH_4_NO_3_ | 16.5 g | 82.5 g |
| KNO_3_ | 19.0 g | 95.0 g |
| KH_2_PO_4_ | 1.7 g | 8.5 g |
| MgSO_4_·7H_2_O | 3.7 g | 18.5 g |
| CaCl_2_·2H_2_O | 4.4 g | 22.0 g |

Dissolve CaCl_2_·2H_2_O before mixing with other components.

Store solutions at 4℃ after autoclaving.

**Table S2 |** The formula of 10×L Microsalts stock solution.

| **Components** | **1 L** | **5 L** |
| --- | --- | --- |
| MnSO_4_·H_2_O* | 16.79 g | 83.95g |
| H_3_BO_3_ | 5.0 g | 25g |
| ZnSO_4_·7H_2_O | 7.5 g | 37.5g |
| KI | 0.75 g | 3.75g |
| Na_2_MoO_4_·2H_2_O | 0.25 g | 1.25g |
| CuSO_4_·5H_2_O | 0.025 g | 0.125g |
| CoCl_2_·6H_2_O | 0.025 g | 0.125g |

Store solutions in dark at 4℃ after sterilization.

* If MnSO_4_·H_2_O is not available for making this solution, any of the following chemicals can be used with the amounts indicated.

| MnSO_4_ | 15.0g | MnSO_4_·4H_2_O | 22.15g | MnSO_4_·7H_2_O | 27.51g |
| --- | --- | --- | --- | --- | --- |

**Table S3 |** The formula of 100×MS FeNaETA stock solution.

| **Components** | **1 L** | **5 L** |
| --- | --- | --- |
| Na_2_EDTA.2H_2_O | 3.725 g | 18.625g |
| FeSO_4_.7H_2_O | 2.785 g | 13.925g |

Store solutions in dark at 4℃ after sterilization.

**Table S4 |** The formula of 1000×MS vitamins (-Glycine) stock solution.

| **Components** | **100 mL** | **200 mL** |
| --- | --- | --- |
| Nicotinic acid | 50 mg | 100 mg |
| Thiamine HCl | 10 mg | 20 mg |
| Pyridoxine HCl | 50 mg | 100 mg |

Store solutions in dark at 4℃ after sterilization.

**Table S5 |** The formula of 25×3AA (Amino Acid) stock solution.

| **Components** | **1 L** | **2 L** |
| --- | --- | --- |
| L-Glutamine | 18.75 g | 37.5 g |
| L-Proline | 3.75 g | 7.5 g |
| L-Asparagine | 2.50 g | 5.0 g |

L-Glutamine (Sigma Aldrich, Catalog No. G-5763) can be dissolved in H_2_O.

Store solution in dark at -20℃ in 40 mLs/200 mLs.

**Table S6 |** The formula of 100 mg/mL of myo-inositol stock solution.

| **Components** | **0.5 L** | **1 L** |
| --- | --- | --- |
| Myo-inositol | 5 g | 10 g |

**Table S7 |** The formula of 10×L7 Macrosalts stock solution.

| **Components** | **1 L** | **5 L** |
| --- | --- | --- |
| NH_4_NO_3_ | 2.5 g | 12.5 g |
| KNO_3_ | 15.0 g | 75.0 g |
| KH_2_PO_4_ | 2.0 g | 10.0 g |
| MgSO_4_·7H_2_O | 3.5 g | 17.5 g |
| CaCl_2_·2H_2_O | 4.5 g | 22.5 g |

Dissolve CaCl_2_·2H_2_O before mixing with other components.

Store solutions at 4℃ after autoclaving.

**Table S8 |** The formula of 200×L vitamins&inositol stock solution.

| **Components** | **1 L** | **2 L** |
| --- | --- | --- |
| Inositol | 40.0 g | 80.0 g |
| Thiamine HCl | 2.0 g | 4.0 g |
| Nicotinic acid | 0.20 g | 0.40 g |
| Pyridoxine HCl | 0.20 g | 0.40 g |
| Ascorbic acid | 0.20 g | 0.40 g |
| Ca-Pantothenate | 0.20 g | 0.40 g |

Store solutions in 50 mL aliquots at -20℃.

**Table S9 |** Summary of the reported work of wheat genetic transformation mediated by particle gun.

| **The target gene plasmid** | **Target Tissue** | **Cultivar** | **Transformation efficiency** | **Gene Function** | **Reference** |
| --- | --- | --- | --- | --- | --- |
| pA25-TaGW2-RNAi | Immature embryos | Shi 4185 | --------- | Influence grain width and weight | Hong et al. (2014) |
| pCOR113 -Yr10 | Immature embryos | Fielder | --------- | Resistance to stripe rust | Liu et al. (2014) |
| pA25-TaERF3 | Immature embryos | Yangmai6 | 0.33% | Enhance salt and drought tolerance | Rong et al. (2014) |
| 35S::ZmPEPC, 35S::ZmPPDK | Immature embryos | Zhoumai19 | --------- | Improve photosynthetic characteristics | Zhang et al. (2014) |
| *pAHC::TcLr19PR1* | embryonic calli | Zhengzhou5389 | 0.58 % | Resistance against leaf rust fungus | Gao et al. (2015) |
| pUbi::TaNAC2-pAHC25 | Immature embryos | Longchun23 | --------- | Increase yield | He et al. (2015) |
| pUbi::TaNFYA-B1-pAHC25 | Immature embryos | Longchun23 | --------- | Increase nitrogen and phosphorus uptake, increase grain yield | Qu et al. (2015) |
| pUbi-TaNF-YB4 | Immature embryos | Gladius | --------- | Improve grain yield | Yadav et al. (2015) |
| pBract302-TaNAC-S | Immature embryos | Cadenza | --------- | Influence leaf senescence | Zhao et al. (2015) |
| pRSS1P:myc-TaCAD12 | Immature embryos | Yangmai 16 | 0.2% | Resistance to sharp eyespot | Rong et al. (2016) |
| pZmRab17::TaDREB3 | Immature embryos | Bobwhite | --------- | Improves drought tolerance | Shavrukov et al. (2016) |
| pA25- myc-TaCPK7-D | Immature embryos | Yangmai16 | 0.25% | Enhance wheat  resistance to sharp eyespot | Wei et al. (2016) |
| pUba-GFP | mature embryos | Fielder, Haruyokoi | 1.39%, 2.28% | No biological function | Hamada1 et al. (2017) |
| pUBI::myc-TaPIMP2 | Immature embryos | Yangmai16 | --------- | Host resistance | Wei et al. (2017) |
| pAHC25-SeCspA, pAHC25-SeCspB | Immature embryos | Kenong199 | --------- | Drought tolerance | Yu et al. (2017) |
| pAHC25-MCS2-DvRGA2 | Not mentioned | Kenong 199 | --------- | Powdery mildew resistance | He et al. (2018) |
| *pUbi:tae-MIR172* | Immature embryos | Kenong199 | --------- | Regulation spike architecture | Liu et al. (2018) |
| pBI220-NP::NLR1-V | Immature embryos | Yangmai158 | --------- | Powdery mildew resistance | Xing et al. (2018) |
| *pUbi*:*mTaCOLD1* | Immature embryos | Kenong199 | --------- | Reduction plant height | Dong et al. (2019) |
| pAHC17-*TaPm3e* | Immature embryos | Bobwhite | --------- | Powdery mildew resistance | Koller et al. (2019) |
| pANDA-b- *TaGNI1* | Immature embryos | Bobwhite | --------- | Inhibit floret development | Sakuma et al. (2019) |
| pAHC17-*TaHRC*-R | Immature embryos | Bobwhite | --------- | FHB resistance | Su et al. (2019) |
| *Glu-1Bx14pro:TuSPR*, *Ubipro:TaSPR*RNAi | Immature embryos | Kenong 199 | --------- | Influence seed storage protein synthesis | Shen et al. (2020) |
| pCAMBIA1301-TaEXPA2 | Immature embryos | CB037 | --------- | Enhance drought stress tolerance | Yang et al. (2020) |
| pAHC25-GmDREB1 | Embryogenic callus | Jimai19, Jimai20 | 3.0%, 0.8% | Enhance drought stress tolerance | Zhou et al. (2020) |
| ubiquitin-TaOGT1b-pMDC32 | Immature embryos | Duster | --------- | Regulation flowering time | Fan et al. (2021) |
| *Glu-1Bx14pro:TuODORANT1*, *Ubipro:TuODORANT1*RNAi | Immature embryos | Kenong 199 | --------- | Influence seed storage protein synthesis | Luo et al. (2021) |
| Ubi-TaTAP46-5A, pWMB006-TaTAP46-5A-RNAi | Immature embryos | Kenong199 | --------- | Influence thousand-kernel weight and kernel size | Zhang et al. (2021) |
| pSHGmTDN1 | Immature embryos | Shi4185, Jimai22 | 1.93%, 156% | Tolerance to drought and low-N stress | Zhou et al. (2022) |
| ubiquitin-*TaCol-B5*-pMDC32 | Immature embryos | Yangmai18 | --------- | Enhances grain yield | Zhang et al. (2022) |

**Supplementary Figures**

**
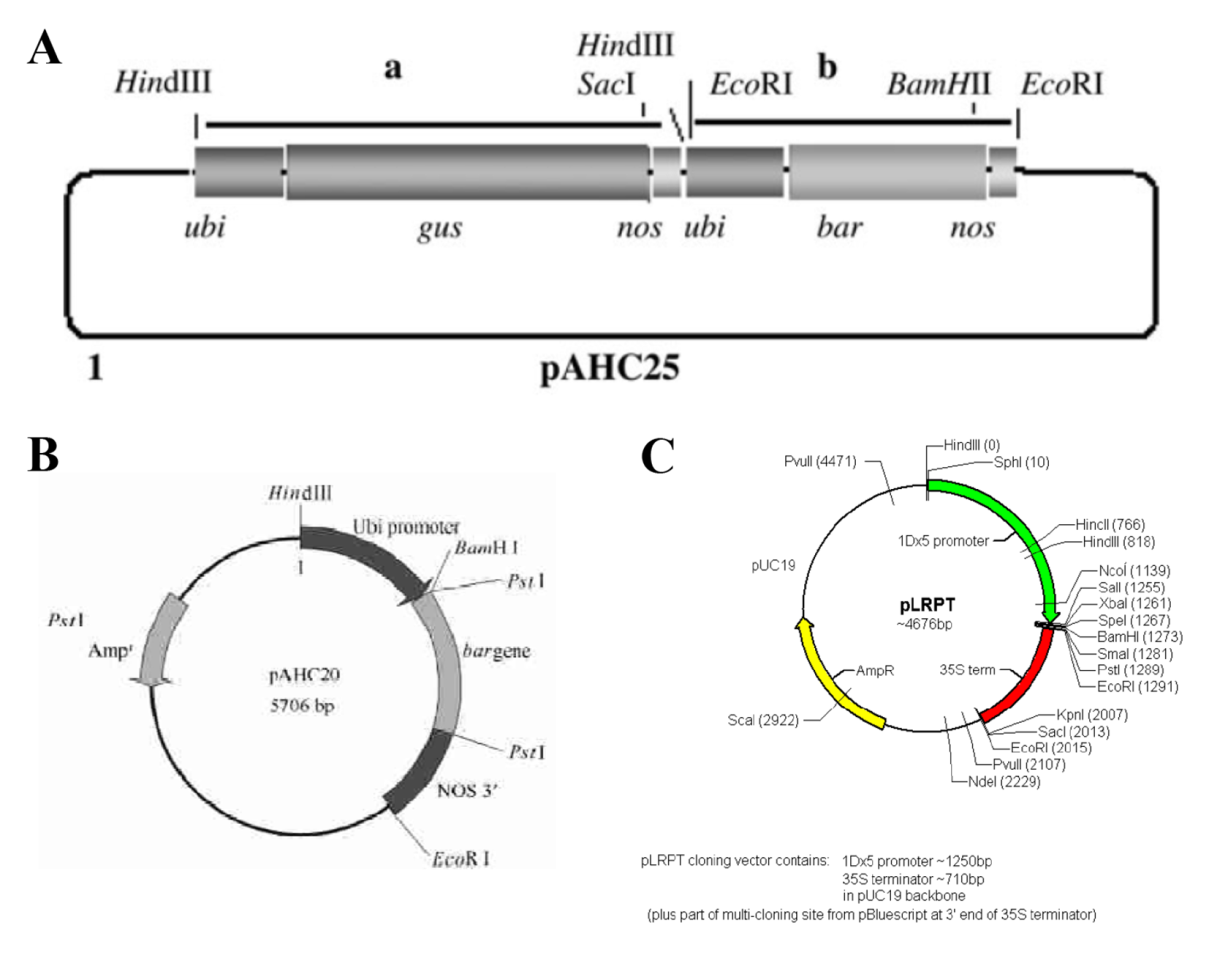
**

**Figure S1** | Diagram of the three representative vectors used for particle bombardment-mediated wheat transformation. **(A)** pAHC25 contains the visible marker gene *uidA* encoding XXX (namely GUS) driven by the constitutive promoter Ubiquitin 1 (*ubi*) from maize and the selectable marker gene bar driven by the *ubi* promoter, too. When the gene of interest (GOI) is to be integrated into pAHC25, the GOI can place the *uidA* gene by double-enzyme digestion and ligation or the expression cassette can be ligated into the vector through single-enzyme digestion and ligation. **(B)** pAHC20 contain the expression cassette of the selectable marker gene bar driven by the maize ubi promoter. **(C)** the pLRPT vector contains the seed-specific promoter *1Dx5* and *35S* terminator with a multiple cloning site containing several restriction-enzyme sites for gene insertion.

**
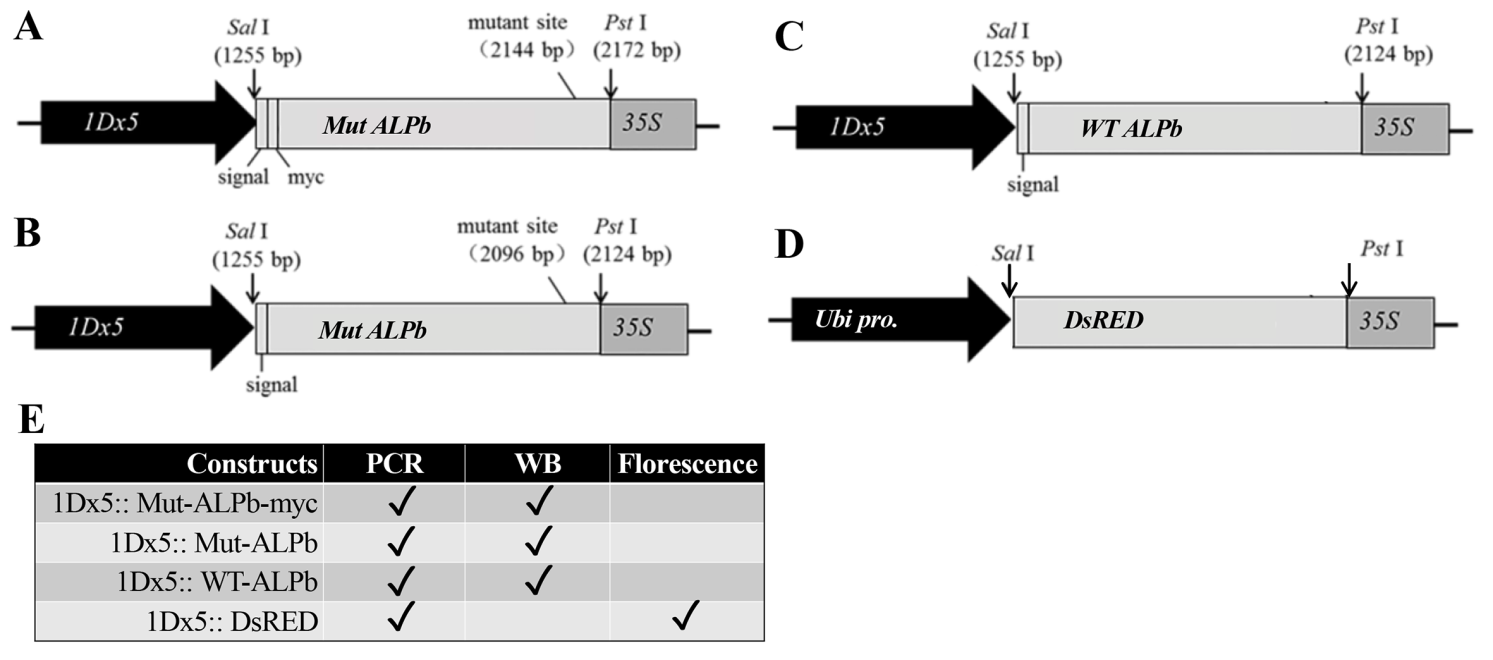
**

**Figure S2** | The diagram of the constructs used for generating transgenic wheat lines is shown in Figure 6 and Figure S3. **(A)** The construct contains the point mutated gene encoding avenin-like b (namely Mut-ALPb) with its signal peptide and myc tag fused in the N-terminal. This Mut-ALPb is driven by the 1Dx5 promoter and 35S terminator. The point mutation site is indicated in the figure. **(B)** The construct contains the gene encoding wild-type ALPb (namely WT-ALPb) driven by the 1Dx5 promoter and 35S terminator. **(C)** The construct contains the point mutated gene encoding Mut-ALPb, driven by the 1Dx5 promoter and 35S terminator. **(D)** The construct contains the visible marker gene DsRED driven by the ubiquitin promoter and 35S terminator. **(E)** The table illustrates different strategies suitable for the identification of transgenic positive plants.

**
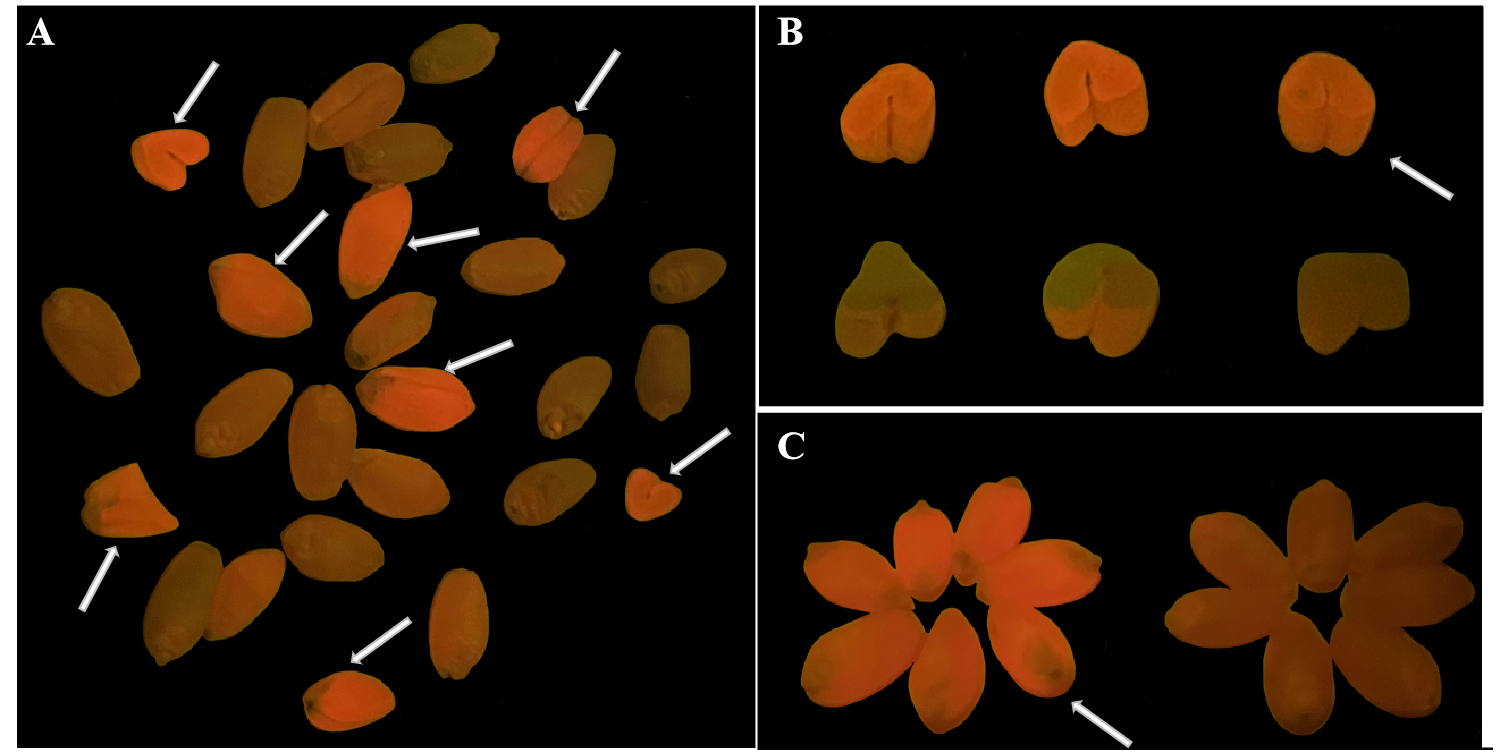
**

**Figure S3** | Identification of transgenic wheat seeds expressing the visible marker DsRED by fluorescent detection with the naked eye. **(A)**, **(B)** and **(C)** are representative pictures of the transgenic seeds harvested from the same spike or the same plants. The seeds with red fluorescence are indicated by white arrowheads.

**Supplementary Notes**

**Note 1**: Besides the reagents for seed disinfection as described in the main text, other reagents have been reported for seed disinfection as follows: (1) 10% (v/v) Domestos (Lever Faberge, UK) (Wang 2016); (2) 5% sodium hypochlorite for 15 min (Wang et al. 2022).

**Note 2**: Some settling of salts may occur during storage for the stock solutions, so the medium should be shaken well before use. The stock solutions stored at −20°C should be effective for more than 12 months without frequent freeze/thawing.

**Note 3**: After autoclaving, 2 × Agargel may be unevenly melted. So before using it for preparing the plates of the medium, 2 × Agargel bottles should be shaken well to avoid non-uniform solidification.

**Note 4**: We suggest that tissue culture media should be prepared as fresh as possible and not to be stored for more than 3 weeks in Petri dishes. However, the medium plates should be prepared a few days in advance to allow any contamination to be detected before use.

**Note 5**: 3% sucrose is used here in the induction medium. This concentration of sucrose is often suitable, while other reports recommend that 9% sucrose could partially plasmolyze the cells during pre-culture and may increase their ability to withstand bombardment.

**Note 6**: We recommend that glufosinate ammonium (PPT) should be used at 4 mg/L with the best inhibitory effects, while its concentration can be adjusted within the range of 2~6 mg/L in the medium. Generally, the selection agent should be used at a concentration known to fully inhibit the growth of non-transformed explants.

**Note 7**: Spermidine is absorbed easily by water so it cannot be weighed on paper.

**Note 8**: Plasmid DNA should be extracted at an appropriate concentration so that the DNA won’t be very dilute.

**Note 9**: We recommend using the wheat plants grown in the field, if possible since they are generally grown more rigorously than those in the greenhouse. We think that the plants grown in a greenhouse, glasshouse, or growth chamber can be used, but they tend to be more variable between plants and spikes.

**Note 10**: Please keep in mind that the developmental rate of immature scutella may vary between cultivars and depends on the environment. The size of the immature scutella appropriate for transformation is also slightly different between cultivars. The size of the developing kernels cannot be used as an indicator for predicting the size of scutella, as both the developmental rates of embryo/scutella and endosperm, respectively, vary between cultivars.

**Note 11**: We suggest that the spikes should be collected when it's not rainy or at least two consecutive sunny days after the rain. Otherwise, the chance of getting an infection will rise. During 12~16 days after anthesis, the embryos develop quickly and could turn to be ready in a few hours after a previous observation.

**Note 12**: Since the immature seeds from the top or bottom spikelets possibly flower and develop at a different time compared with the seeds from the middle part of the same spike, we recommend that only the seeds collected from the middle spikelets are used for transformation to ensure the high quality and uniformity of the immature scutella. We suggest that the inner caryopses should be avoided as they tend to contain smaller embryos.

**Note 13**: The entire process of wheat genetic transformation should be performed aseptically.

**Note 14**: After HgCl_2_ sterilization for 8 minutes, we emphasize at least three changes of water to fully get rid of the residual HgCl_2_ to avoid its harm to immature scutella’s growth.

**Note 15**: We suggest that a full plate of unsterilized seeds can be dispensed into 5~6 plates after seed disinfection. Too many disinfected seeds in one plate might increase the chance of getting an infection as more water and milky starch will be cut and spread in the plate. When getting rid of the excess water during the seed disinfection step, use tweezers to keep the seeds and get rid of water out of the flask.

**Note 16**: We recommend that the disinfected seeds should be used within 24 hours for the isolation of immature scutella.

**Note 17**: As shown in Figure 2D, we suggest that the scalpel blade should first cut the caryopses on top of the axis. Once the caryopses and the axis has grown beneath are cut off, the scutellum can be easily taken out by using the tip of the scalpel blade without touching the endosperm.

**Note 18**: Within the circle where isolated scutella should be placed (**Figure 2E**), a least of 20~30 immature scutella can be placed. We recommend placing 45~50 scutella to fully use the space while not to being too crowded after the preculture of the scutella. A maximum of 70~80 scutella can be placed within the circle, however, after preculture the scutella will grow and get overlapped with each other, thus leading to a higher chance of getting the infection and spreading between the scutella. Another disadvantage of overcrowded scutella is that the scutella placed in the peripheral areas may accept fewer gold particles than those in the central area, as the particle gun fires most gold particles within the central circular area (in diameter of approximately 2 cm) of a Petri dish.

**Note 19**: Preculture of the isolated scutella can be lasted for 12 to 24 hours but is not recommended for more than 48 hours. The preculture phase is recommended as it allows the tissue to recover from the isolation before being subject to particle bombardment.

**Note 20:** 5 µg of DNA (in the form of either plasmids or linear DNA fragments) is the standard amount for coating with gold particles. If using multiple plasmids or linear DNA fragments for co-transformation, equimolar quantities of each DNA molecule should be used with a total amount of 5 µg. Excess DNA may cause clumping of gold particles. Importantly, different ratios may skew the probability of co-transformation of the genes in these DNA molecules in transgenic plants. Theoretically, linear DNA fragments likely lead to higher transformation efficiency when compared with those using plasmid DNAs with the same set of bombardment parameters, as more DNA molecules are coated with the gold particles given the 5 µg of total DNA amount.

**Note 21:** In the preparation of gold particles, the gold powder particles should be completely suspended to avoid uneven gold powder particles in each tube dispensing. The clumped gold particles cannot be removed during later suspension steps. In steps 17 and 18, the particles should be completely resuspended by scraping the side of the tube with the pipette tip to remove clumps. Vortex will not help re-suspension. 25 μL of gold is sufficient for seven shots, while the gold and other components can be scaled accordingly to the number of shots.

**Note 22:** The effects of CaCl_2_ and spermidine are to bind, stabilize, and precipitate the DNA. DNA precipitates onto the gold particles in a very short period, and the CaCl_2_ and spermidine are therefore mixed first to ensure that the coating is as even as possible.

**Note 23:** The whole process of preparing DNA-coated gold particles should be finished rapidly (recommended within 30 minutes) without pause. DNA-coated gold particles can be only kept on ice for no longer than one hour.

**Note 24:** Rupture discs of 450, 650, 900, 1,100, 1,350, 1,550, 1,800, 2,000, and 2,200 psi are available for purchase. We used a 1,100-psi rupture disc for most cases, while other reports also found a 900-psi rupture disc optimal for wheat transformation. Rupture discs with higher or lower psi will result in successful transformation but with lower efficiency.

**Note 25: [linen 295]** The following control plates should be included: (1) unbombarded scutella to monitor the development and regeneration of the donor tissue; (2) the scutella bombarded with gold (no DNA) and unselected to determine the tissue culture status after bombardment; (3) the scutella bombarded with gold (no DNA) and subject to selection to monitor the effectiveness of the selection.

**Note 26:** The present protocol is based on the PDS-1000/He particle gun (Bio-Rad), while other manufacturers also produce similar instruments with different consumables and parameters, such as GJ-1000 high-pressure gas gene delivery system (Scientz, China). The detailed parameters and procedures for particle bombardment may need optimization.

**Note 27:** Sterilization of the rupture discs longer than 10 min may lead to the laminate layers separated from the discs.

**Note 28:** If the Fire switch is released too early, the rupture disc will not burst.

**Note 29:** One possible reason for lowered burst pressure of the rupture disc could be that the retaining cap is not tightly installed.

**Note 30:** The stopping screens and macrocarriers need to be sonicated to remove the remaining DNA to prevent adhering DNA from carrying over to future bombardments. After the shot, a good mesh indentation should be seen on the macrocarrier as it hit the stopping screen if the shot has been operated correctly.

**Note 31:** We suggest that in the first few days after the scutella have been transferred to the induction medium, they should be checked every day for any contamination. Once contaminations are observed, the other uncontaminated, un-neighboring explants should be transferred immediately to a new plate of the induction medium to save them from contamination. After the first 5 to 7 days, checking for contamination should be less frequent.

**Note 32:** When plating the differentiation medium, the plates should be thicker than those of the induction medium. Based on our experience, we suggest that 1 L of the 1× induction medium should be poured into 30~33 plates (9-cm Petri dish), while 1 L of the 1×differentiation medium should be poured into ~25 plates. One liter of the 1× rooting medium should be poured into 28~30 culture cups.

**Note 33:** The two alternate routes for regeneration and selection have respective advantages and disadvantages. Route 1 allows the embryogenic callus to fully grow, respond to the phytohormones in the medium, and differentiate, whereas it takes more plates of the medium and the workload of tissue culture. Since the regenerated plantlets are not selected, a large number of regenerated plantlets will enter the rooting stage, which will occupy a relatively larger space. Moreover, the selection during the rooting stage could take longer time and be less effective as the regenerated seedlings are growing bigger. By contrast, route 2 employs selection in the regeneration stage, likely lowering the probability of obtaining “escape plants” and taking less space and workload compared to route 1. Thus, route 2 is suitable for large-scale transformation experiments.

**Note 34:** When the regenerated calli are transferred between plates, we suggest that the calli should not be separated into smaller pieces, otherwise they need to be tracked accordingly when labeling the corresponding plates. If some calli are bigger than others, the number of calli in that plate should be decreased to prevent overcrowding.

**Note 35:** We suggest that when transferring the regenerated plantlets onto the rooting medium, the attached callus should be half buried into the medium by using the tip of a pair of tweezers to ensure that the callus could efficiently uptake the nutrients and respond to the phytochromes from the plate.

**Note 36:** Tissue grinding can be performed by using either pestle and mortar, plastic grinding sticks o r high-throughput tissue grinding machines.

**Note 37:** Several pairs of primers for the detection of construct components (such as *35S* terminator, *bar* and *uidA* genes) are listed as follows:

| **Target**  **site** | **Primers** | **Sequence ( 5’ - 3’)** | **Tm** | **Product length** |
| --- | --- | --- | --- | --- |
| 35S terminator | CaMV 35S-F | CGCTGAAATCACCAGTCT | 59℃ | 417 bp |
|  | CaMV 35S-R | TCCTTCCTTCCGTCCACT |  |  |
| *bar* | Bar-F | CTGCACCATCGTCAACCACTACATC | 55℃ | 460 bp |
|  | Bar-R | AGCTGCCAGAAACCCACGTCA |  |  |
| *uidA* | Gus-F | AGTGTACGTATCACCGTTTGTGTGAAC | 63℃ | 1056 bp |
|  | Gus-R | ATCGCCGCTTTGGACATACCATCCGTA |  |  |
| *uidA* | Gus-F1 | TGCTCTACACCACGCCGAAC | 65℃ | 636 bp |
|  | Gus-R1 | CTTGCTGAGTTTCCCCGTTG |  |  |
| *DsRED* | DsRed-s1 | ATGGCCTCCTCCGAGAACGT | 66℃ | 804 bp |
|  | DsRed-a1 | CATCGCAAGACCGGCAACA |  |  |

**Supplementary References**

Dong, H. X., Yan, S. L., Liu, J., Liu, P., and Sun, J. Q.  (2019). *TaCOLD1* defines a new regulator of plant height in bread wheat. *Plant Biotechnol. J*. 17, 687–699. doi: 10.1111/pbi.13008

Fan, M., Miao, F., Jia, H. Y., Li, G. Q., Powers, C., Nagarajan, R., et al. (2021). O-linked N-acetylglucosamine transferase is involved in fine regulation of flowering time in winter wheat. *Nat. Commun*. 12, 2303. doi: 10.1038/s41467-021-22564-8

Gao, L., Wang, S., Li, X. Y., Wei, X. J., Zhang, Y. J., Wang, H. Y., et al. (2015). Expression and functional analysis of a pathogenesisrelated protein 1 gene, *TcLr19PR1*, involved in wheat resistance against leaf rust fungus. *Plant Mol. Biol. Rep*. 33, 797–805. doi: 10.1007/s11105-014-0790-5

Hamada, H., Linghu, Q., Nagira, Y., [Miki](https://pubmed.ncbi.nlm.nih.gov/?term=Miki+R&cauthor_id=28904403) , R., [Taoka](https://pubmed.ncbi.nlm.nih.gov/?term=Taoka+N&cauthor_id=28904403), N., and [Imai](https://pubmed.ncbi.nlm.nih.gov/?term=Imai+R&cauthor_id=28904403), R. (2017). An in planta biolistic method for stable wheat transformation. *Sci. Rep*. 7, 11443. doi: 10.1038/s41598-017-11936-0

He, H. G., Zhu, S. Y., Zhao, R. H., Jiang, Z. N., Ji, Y. Y., Ji. J., et al. (2018). *Pm21*, encoding a typical CC-NBS-LRR protein, confers broad-spectrum resistance to wheat powdery mildew disease. *Mol. Plant* 11, 879–882. doi :10.1016/j.molp.2018.03.004

He, X., Qu, B. Y., Li, W. J., Zhao, X. Q., Teng, W., Ma, W. Y., et al. (2015). The nitrate-inducible NAC transcription factor *TaNAC2-5A* controls nitrate response and increases wheat yield. *Plant Physiol.* 169, 1991–2005. doi: 10.1104/pp.15.00568

Hong, Y. T., Chen, L. F., Du, L. P., Su, Z. Q., Wang, J. F., Ye, X. G., et al. (2014). Transcript suppression of *TaGW2* increased grain width and weight in bread wheat. *Funct. Integr. Genomics* 14, 341–349. doi: 10.1007/s10142-014-0380-5

Koller, T., Brunner, S., Herren, G., Sanchez-Martin, J., and Hurni, S. (2019). Field grown transgenic Pm3e wheat lines show powdery mildew resistance and no ftness costs associated with high transgene expression. *Transgenic Res*. 28, 9–20. doi: 10.1007/s11248-018-0099-5

Liu, P., Liu, J., Dong, H. X., and Sun, J. Q. (2018). Functional regulation of Q by microRNA172 and transcriptional co-repressor TOPLESS in controlling bread wheat spikelet density. *Plant Biotechnol. J*. 16, 495–506. doi: 10.1111/pbi.12790

Liu, W., Frick, M., Huel R., Nykiforuk, C. L., Wang, X. M., Gaudet, D. A., et al. (2014). The stripe rust resistance gene *Yr10* encodes an evolutionary-conserved and unique CC-NBS-LRR sequence in wheat. *Mol. Plant* 7, 1740–1755. doi: 10.1093/mp/ssu112

Luo, G. B., Shen, L. S., Song, Y. H., Yu, K., Ji, J. J., Zhang, C., et al. (2021). The MYB family transcription factor *TuODORANT1* from *Triticum urartu* and the homolog *TaODORANT1* from *Triticum aestivum* inhibit seed storage protein synthesis in wheat. *Plant Biotechnol. J*. 19, 1863-1877. doi: 10.1111/pbi.13604

Qu, B.Y., He, X., Wang, J., Zhao, Y. Y., Teng, W., Shao, A., et al. (2015). A wheat CCAAT boxbinding transcription factor increases the grain yield of wheat with less fertilizer input. *Plant Physiol*. 167, 411–423. doi: 10.1104/pp.114.246959

Rong, W., Luo, M. Y., Shan, T. L, Wei, X. N., Du, L. P., Xu, H. J., et al. (2016). A Wheat cinnamyl alcohol dehydrogenase *TaCAD12* contributes to host resistance to the sharp eyespot disease. *Front. Plant Sci*. 7, 1723. doi: 10.3389/fpls.2016.01723

Rong, W., Qi, L., Wang, A. Y., Ye, X. G., Du, L. P., Liang, H. X., et al. (2014). The ERF transcription factor *TaERF3* promotes tolerance to salt and drought stresses in wheat. *Plant Biotechnol J*. 12, 468–479. doi: 10.1111/pbi.12153

Sakuma, S., Golan, G., Guo, Z. F., Ogawa, T., Tagiri, A., Sugimoto, K., et al. (2019). Unleashing foret fertility in wheat through the mutation of a homeobox gene. *Proc. Natl. Acad. Sci. USA*. 116, 5182–5187. doi: 10.1073/pnas.1815465116.

Shavrukov, Y., Baho, M., Lopato, S., and Langridge, P. (2016). The *TaDREB3* transgene transferred by conventional crossings to different genetic backgrounds of bread wheat improves drought tolerance. *Plant Biotechnol. J*. 14, 313–322. doi: 10.1111/pbi.12385

Shen, L. S., Luo, G. B., Song, Y. H., Xu, J. Y., Li, J. J., Zhang, C., et al. (2020). A novel NAC family transcription factor *SPR* suppresses seed storage protein synthesis in wheat. *Plant Biotechnol. J.* 19, 992–1007. doi: 10.1111/pbi.13524

Su, Z., Bernardo, A., Tian, B., Chen, H., Wang, S., Ma, H. X., et al. (2019). A deletion mutation in *TaHRC* confers *Fhb1* resistance to Fusarium head blight in wheat. *Nat. Genet.* 51, 1099–1105. doi: 10.1038/s41588-019-0425-8

Wei, X. N., Shan, T. L., Hong, Y. T., Xu, H. J., Liu, X., and Zhan, Z. Y. (2017). *TaPIMP2*, a pathogen-induced MYB protein in wheat, contributes to host resistance to common root rot caused by Bipolaris sorokinian. *Sci. Rep*. 7, 1754. doi: 10.1038/s41598-017-01918-7

Wei, X. N., Shen, F. D., Hong, Y. T., Rong, W., Du, L. P., Liu, X., et al. (2016). The wheat calcium-dependent protein kinase *TaCPK7-D* positively regulates host resistance to sharp eyespot disease. *Mol. Plant Pathol*. 17, 1252–1264. doi: 10.1111/mpp.12360

Xing, L. P., Hu, P., Liu, J. Q., Witek, K., Zhou, S., Xu, J. F., et al. (2018). *Pm21* from *Haynaldia villosa* encodes a CC-NBS-LRR protein conferring powdery mildew resistance in wheat. *Mol. Plant* 11, 874–878. doi: 10.1016/j.molp.2018.02.013

Yadav, D., Shavrukov, Y., Bazanova, N., Chirkova, L., Borisjuk, N., Kovalchuk, N., et al. (2015). Constitutive overexpression of the *TaNF-YB4* gene in transgenic wheat signifcantly improves grain yield. *J. Exp. Bot*. 66, 6635–6650. doi: 10.1093/jxb/erv370

Yang, J. J., Zhang, G. Q., An, J., Li, Q. X., Chen, Y. H., Zhao, X. Y., et al. (2020). Expansin gene *TaEXPA2* positively regulates drought tolerance in transgenic wheat (*Triticum aestivum* L.). *Plant Sci*. 298, 119056. doi: 10.1016/j.plantsci.2020.110596

Yu, T. F., Xu, Z. S., Guo, J. K., Wang, Y. X., [Abernathy](https://pubmed.ncbi.nlm.nih.gov/?term=Abernathy+B&cauthor_id=28281578), B., Fu, J. D., et al. (2017). Improved drought tolerance in wheat plants overexpressing a synthetic bacterial cold shock protein gene *SeCspA*. *Sci. Rep*. 7, 44050. doi: 10.1038/srep44050

Zhang, H. F., Xu, W. G., Wang, H. W., Hu, L., Li, Y., Qi, X. L., et al. (2014). Pyramiding expression of maize genes encoding phosphoenolpyruvate carboxylase (PEPC) and pyruvate orthophosphate dikinase (PPDK) synergistically improve the photosynthetic characteristics of transgenic wheat. *Protoplasma* 251, 1163–1173. doi: 10.1007/s00709-014-0624-1

Zhang, X. Y., Jia, H. Y., Li, T., Wu, J. Z., Nagarajan, R., Lei, L., et al., (2022). *TaCol-B5* modifies spike architecture and enhances grain yield in wheat. *Science* 376, 180–183. doi: 10.1126/science.abm0717

Zhang, Y. F., Li, T., Geng, Y. K., Wang, Y. M., Liu, Y. C., Li, H. F., et al. (2021). Identification and development of a KASP functional marker of *TaTAP46* associated with kernel weight in wheat (*Triticum aestivum*). *Plant Breed*. 140, 585–594. doi: 10.1111/pbr.12922

Zhao, D., Derkx, A. P., Liu, D. C., Buchner, P., and Hawkesford, M. J. (2015). Overexpression of a NAC transcription factor delays leaf senescence and increases grain nitrogen concentration in wheat. *Plant Biol*. 17, 904–913. doi: 10.1111/plb.12296

Zhou, Y. B., Chen, M., Guo, J. K., Wang, Y. X., Min, D. H., Jiang, Q. Y., et al. (2020). Overexpression of soybean *DREB1* enhances drought stress tolerance of transgenic wheat in the field. *J. Exp. Bot.* 71, 1842–1857. doi: 10.1093/jxb/erz569

Zhou, Y. B., Liu, J., Guo, J. K., Wang, Y. X., Li, H. T., Chu, X. S., et al. (2022). *GmTDN1* improves wheat yields by inducing dual tolerance to both drought and low-N stress. *Plant Biotechnol. J*. doi: 10.1111/pbi.13836
